# A novel protein encoded by circular SMO RNA is essential for Hedgehog signaling activation and glioblastoma tumorigenicity

**DOI:** 10.1186/s13059-020-02250-6

**Published:** 2021-01-14

**Authors:** Xujia Wu, Songhua Xiao, Maolei Zhang, Lixuan Yang, Jian Zhong, Bo Li, Fanying Li, Xin Xia, Xixi Li, Huangkai Zhou, Dawei Liu, Nunu Huang, Xuesong Yang, Feizhe Xiao, Nu Zhang

**Affiliations:** 1 Department of Neurosurgery, Institute of Precision Medicine, The First Affiliated Hospital of Sun Yat-sen University; Guangdong Provincial Key Laboratory of Brain Function and Disease, Guangzhou, 510080 Guangdong China; 2 Department of Neurology, The Sun Yat-sen Memorial Hospital of Sun Yat-sen University, Guangzhou, 510000 Guangdong China; 3 Department of Biochemistry and Molecular Biology, Zhongshan School of Medicine, Sun Yat-sen University, Guangzhou, 510080 Guangdong China; 4 Department of Pathology, The First Affiliated Hospital of Sun Yat-sen University, Guangzhou, 510080 Guangdong China; 5 Department of Scientific Research Section, The First Affiliated Hospital of Sun Yat-sen University, Guangzhou, 510080 Guangdong China

**Keywords:** Glioblastoma, Circular RNA, Novel protein, Brain cancer stem cells, Hedgehog pathway

## Abstract

**Background:**

Aberrant activation of the Hedgehog pathway drives tumorigenesis of many cancers, including glioblastoma. However, the sensitization mechanism of the G protein-coupled-like receptor smoothened (SMO), a key component of Hedgehog signaling, remains largely unknown.

**Results:**

In this study, we describe a novel protein SMO-193a.a. that is essential for Hedgehog signaling activation in glioblastoma. Encoded by circular SMO (circ-SMO), SMO-193a.a. is required for sonic hedgehog (Shh) induced SMO activation, via interacting with SMO, enhancing SMO cholesterol modification, and releasing SMO from the inhibition of patched transmembrane receptors. Deprivation of SMO-193a.a. in brain cancer stem cells attenuates Hedgehog signaling intensity and suppresses self-renewal, proliferation in vitro, and tumorigenicity in vivo. Moreover, circ-SMO/SMO-193a.a. is positively regulated by FUS, a direct transcriptional target of Gli1. Shh/Gli1/FUS/SMO-193a.a. form a positive feedback loop to sustain Hedgehog signaling activation in glioblastoma. Clinically, SMO-193a.a. is more specifically expressed in glioblastoma than SMO and is relevant to Gli1 expression. Higher expression of SMO-193a.a. predicts worse overall survival of glioblastoma patients, indicating its prognostic value.

**Conclusions:**

Our study reveals that SMO-193a.a., a novel protein encoded by circular SMO, is critical for Hedgehog signaling, drives glioblastoma tumorigenesis and is a novel target for glioblastoma treatment.

## Background

As a key regulator for fate determination of embryonic stem cells but quiescent in adult cells, aberrant HH pathway activation was frequently observed in many human cancers such as medulloblastoma, basal cell carcinoma (BCC), and GBM [1–3]. The core components of HH signaling including the HH ligands [Sonic hedgehog (Shh), Indian hedgehog (Ihh), and Desert hedgehog (Dhh)], the patched transmembrane receptors (PTCH) 1 and 2, the G protein-coupled-like receptor smoothened (SMO), and the glioma-associated oncoproteins Gli1, Gli2, and Gli3 [4]. HH-mediated signaling transduction was activated through the binding of HH to PTCH and de-repressing SMO, which released Gli1 transcriptional factor from human Suppressor-of-Fused (SUFU), allowing Gli1 nucleus-translocation and gene-expression regulation. Although PTCH did not suppress SMO by direct interaction, structural and chemical biology studies demonstrated that cholesterol modification was required for releasing SMO from PTCH inhibition upon Shh stimulation and cholesterol was considered as the endogenous ligand of SMO [5]. Nevertheless, the detail mechanism of releasing SMO from PTCH inhibition, which is the key to understand HH signaling, remains largely unknown [6].

HH signaling stimulated the transcription of a panel of oncogenic proteins, including Bmi1, Myc, and VEGFA that promoted cancer cell survival, invasion, and angiogenesis [7–9]. HH signaling inhibition attenuated the self-renewal and tumorigenicity of patient-derived brain cancer stem cells (CSCs) [10, 11]. As the hub of the HH signaling, SMO mutation was highly enriched in adult medulloblastoma [2]. SMO overexpression was also seen in glioma, and its expression level correlated with tumor grade and patient prognosis [12]. Previous study demonstrated that HH signaling activation was present in a subset of GBM tumors, and SMO inhibition was effective in glioma lines highly expressing Gli1, indicating HH signaling is likely to be a driver in a subset of GBMs [13]. Thus, targeting SMO is a rational strategy for cancers with abnormal HH signaling status [14]. Indeed, vismodegib, a SMO-specific inhibitor, is a promising therapeutic approach for cancer treatment in BCC and medulloblastoma [15, 16], but the effectiveness remains to be determined in GBM [17].

Circular RNAs (circRNAs) are covalently closed RNA transcripts that widely express in eukaryotes and involve in multiple physio- and pathological processes [18]. Abnormal expression of circRNAs regulate the processes of proliferation, invasion, and angiogenesis in GBM and might serve as potential novel biomarkers or therapeutic targets [19]. CircRNAs were generally considered as non-coding RNA (ncRNA) [18], exerting their functions during gene regulation mainly as micro RNA sponge or protein scaffold [20]. Recently, we and others have reported protein-coding circRNAs, unraveled the hidden functions of circRNAs [21, 22]. CircRNAs encoded proteins usually played auxiliary roles to their linear counterparts and defined the fine-tune system of different biological processes [23]. CircRNA dysregulation is frequently seen in cancers including GBM, raising the hypothesis that imbalanced expression of circRNA-encoded proteins could contribute to tumorigenesis and tumor development [24]. Accordingly, given the unusual expression pattern of certain circRNAs in human malignancies, circRNA-encoded proteins could provide specific targets for cancer diagnosis and treatment.

In this study, we sought to identify circRNA-encoded novel modulators in HH signaling activated GBM. We specifically described the identification of SMO-193a.a. encoded by circ-SMO. We then functionally validated the critical role of SMO-193a.a. in HH signaling and its potential clinical implications for GBM treatment.

## Results

### Circ-SMO expression is enriched in CSCs and GBM

**Fig. 1:**
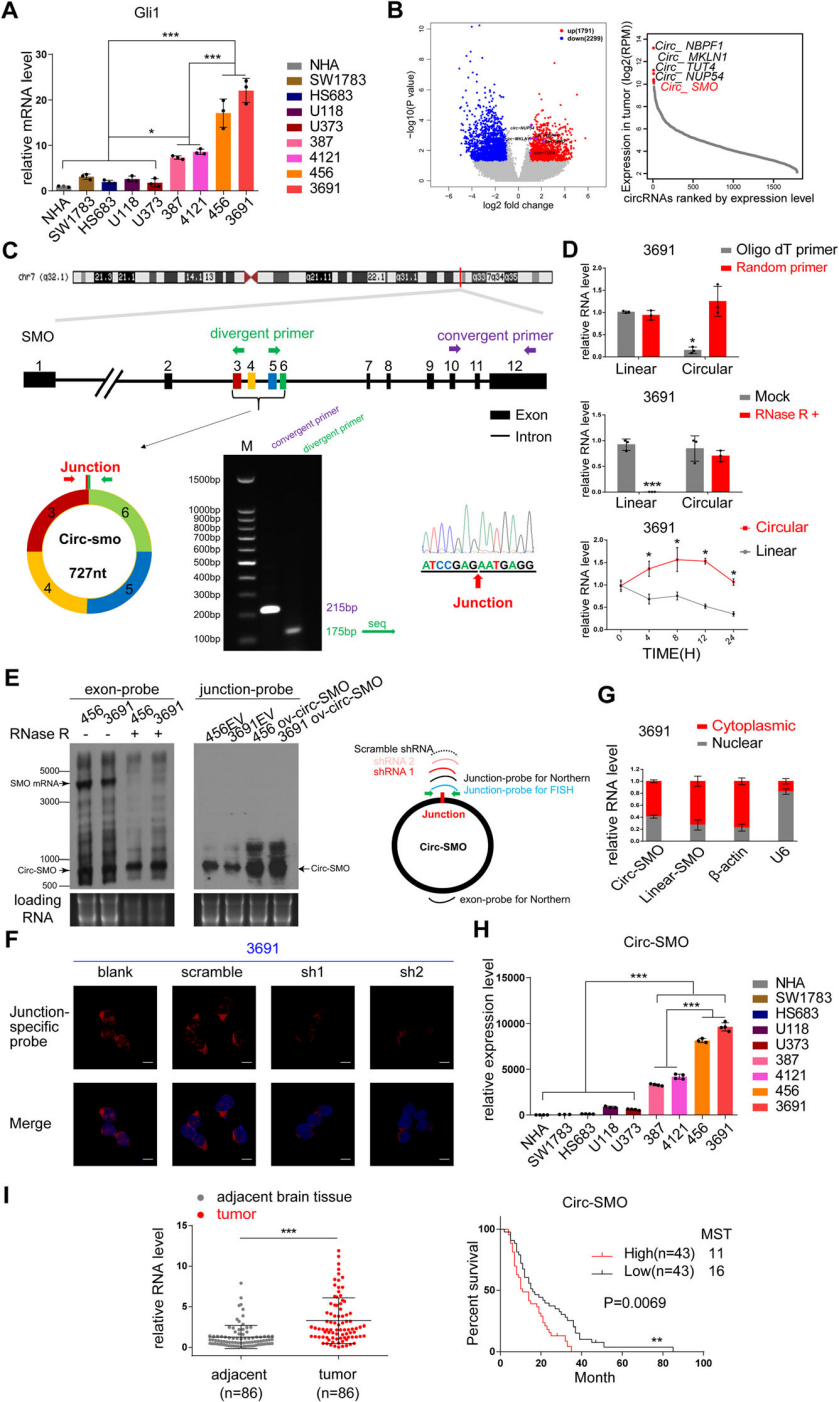
circ-SMO is highly expressed in CSCs and GBM. **a** Gli1 mRNA level in NHA, established GBM cell lines and CSCs. **b** A total of 12 pairs of GBM and NB samples were subjected to RNA-seq and CIRIquant analysis. Left, differentially expressed circRNAs (*p* < 0.01 and fold change > 2) with circBase annotation. In total, 1791 circRNAs were upregulated in GBM; 2299 circRNAs were downregulated in GBM. Right, top five highly expressed circRNAs are listed between GBM and NB. **c** Upper, exons 3–6 of *SMO* formed circ-SMO. Lower left, PCR products of linear SMO and circ-SMO using convergent or divergent primers. Lower right, Sanger sequencing of circ-SMO junction sequences. **d** Circ-SMO characters in 3691 CSC. Upper, qPCR of linear or circ-SMO from oligo dT primers and random primers reversely transcripted cDNA. Middle, qPCR of linear SMO or circ-SMO after RNase R treatment. Lower, half-life of linear SMO or circ-SMO. **e** Left, Northern blotting of circ-SMO and SMO mRNA transcripts by hybridization with exon 4 probes in the absence or presence of RNase R treatment in 456 and 3691 CSCs. Right, junction-specific probe was used to evaluate circ-SMO levels with or without circ-SMO overexpression in indicated cells. Illustration shows the targets of circ-SMO probe, circ-SMO junction shRNAs (referred as sh1 and sh2), and scramble shRNA. **f** Fluorescence in situ hybridization (FISH) of circ-SMO in 3691 CSC with indicated modifications. Bars, 10 μm. **g** Cell fraction qPCR in 3691 CSC. β-actin and U6 were used as cytoplasmic or nuclear markers. **h** Relative expression level of circ-SMO in different cell lines. **i** Left, relative expression level of circ-SMO in 86 GBM patients and their paired adjacent NB tissues. Right, Survival analysis of 86 GBM patients based on circ-SMO expression. The median score of relative expression levels in tumor tissues based on RT-qPCR was used as the cutoff to define “high” or “low” Circ-SMO expression. MST, median survival time. Lines show the mean ± SD. *p < 0.05, **p < 0.01, ***p < 0.001. In **a**, **c**, **d**, **f**, **g**, **h**, **i**, Data are representative from at least three experiments with similar results

To identify differential HH signaling status in glioma, we enrolled a panel of different brain tumor cells including SW1783, HS683 (anaplastic astrocytoma lines), U118, U373 (GBM lines), and 387, 4121, 456, and 3691(CSC lines). Normal human astrocyte (NHA) was used as normal control. Gli1 mRNA expression is a reliable marker for HH signaling activation [25]. Thus, we screened Gli1 mRNA level in above cells to assess HH signaling status. We found that HH signaling was considerably higher activated in CSCs compared with that in glioma cell lines and normal control cells (Fig. 1a). To explore potential circRNA candidates involved in HH signaling activation, we next performed RNA-seq and CIRIquant analysis [26] in twelve GBM samples and their paired normal brain tissues (NB). A total of 76,878 circRNAs were identified and matched in circBase [27] (PRJNA525736) (Fig. 1b, Additional file 1: Fig. S1A). We annotated these identified circRNA candidates using the ensemble database [28]. Most of the identified circRNAs were originated from protein-coding exons and others were aligned with introns, 5′-UTR, 3′-UTR, or antisense sequences (Additional file 1: Fig. S1A). The majority of the identified circRNAs were 300~500 nt in length, which was consistent to our previous report (Additional file 1: Fig. S1B). We identified 1791 highly expressed circRNAs in GBM compared with NB, while 2299 circRNAs were downregulated [false discovery rate (FDR) < 0.05 and fold change > 2] (Fig. 1b, left). Of these differentiated expressed circRNAs, circ-SMO (hsa_circ_0001742) was the top hit circRNA that were generated from HH signaling component genes. Notably, circ-SMO was also ranked top five [transcripts per million (TPM) 1163.2] of all highly expressed circRNAs in GBM compared with that in NB (Fig. 1b, right; Additional file 2: Table S1). Given the central role of SMO played in HH signaling and the high expression level of circ-SMO, we then focused on circ-SMO for next-step investigation.

Circ-SMO was predicted to be formed from exon 3–6 of *SMO* gene [27] (Fig. 1c, upper). We used divergent and convergent primers to perform PCR in 3691 CSC, and followed by Sanger sequencing to confirm the predicted circular junction of circ-SMO [27] (Fig. 1c, lower). Using junction-specific primers, we only amplified circ-SMO in random primer reverse-transcripted but not in oligo dT reverse-transcripted cDNA, and supported the circular form of circ-SMO (Fig. 1d, upper). Compared with SMO mRNA, circ-SMO was more resistant to RNase R digestion (Fig. 1d, middle) and had a longer half-life (Fig. 1d, lower). Using exon probes which designed to recognize both SMO RNA and circ-SMO, we detected both the ~ 700 nt circ-SMO and linear SMO RNA in northern blot from two CSCs (Fig. 1f, left). Treatment of RNase R did not alter the circ-SMO level, while linear SMO level reduced dramatically (Fig. 1e, left). Using junction probe which was designed only for circ-SMO, we also detected endogenous circ-SMO in above CSCs, and overexpression circ-SMO by plasmid (OV-circ-SMO) transfection could elevate circ-SMO expression (Fig. 1e, right). To determine circ-SMO localization, we performed fluorescence in situ hybridization (FISH) in 3691 CSC. Junction-specific probe for circ-SMO and two sh-circ-SMO RNAs (referred as sh1 and sh2 hereafter) were used to confirm the specificity. Circ-SMO mainly displayed a cytoplasmic localization, which was further validated by cell fraction qPCR in 3691 CSC endogenously (Fig. 1f, g). Using junction-specific primers, we further demonstrated that circ-SMO was highly expressed in CSCs compared with that in NHA and GBM cell lines (Fig. 1h). Circ-SMO is also highly expressed in GBM clinical samples compared with that in paired NB and its expression level predicted worse prognosis in a cohort of 86 GBM patients (Fig. 1i). These results suggested that circ-SMO is enriched in several CSCs and may contribute to HH signaling activation in GBM.

### Circ-SMO encodes a novel protein in CSCs and GBM

**Fig. 2:**
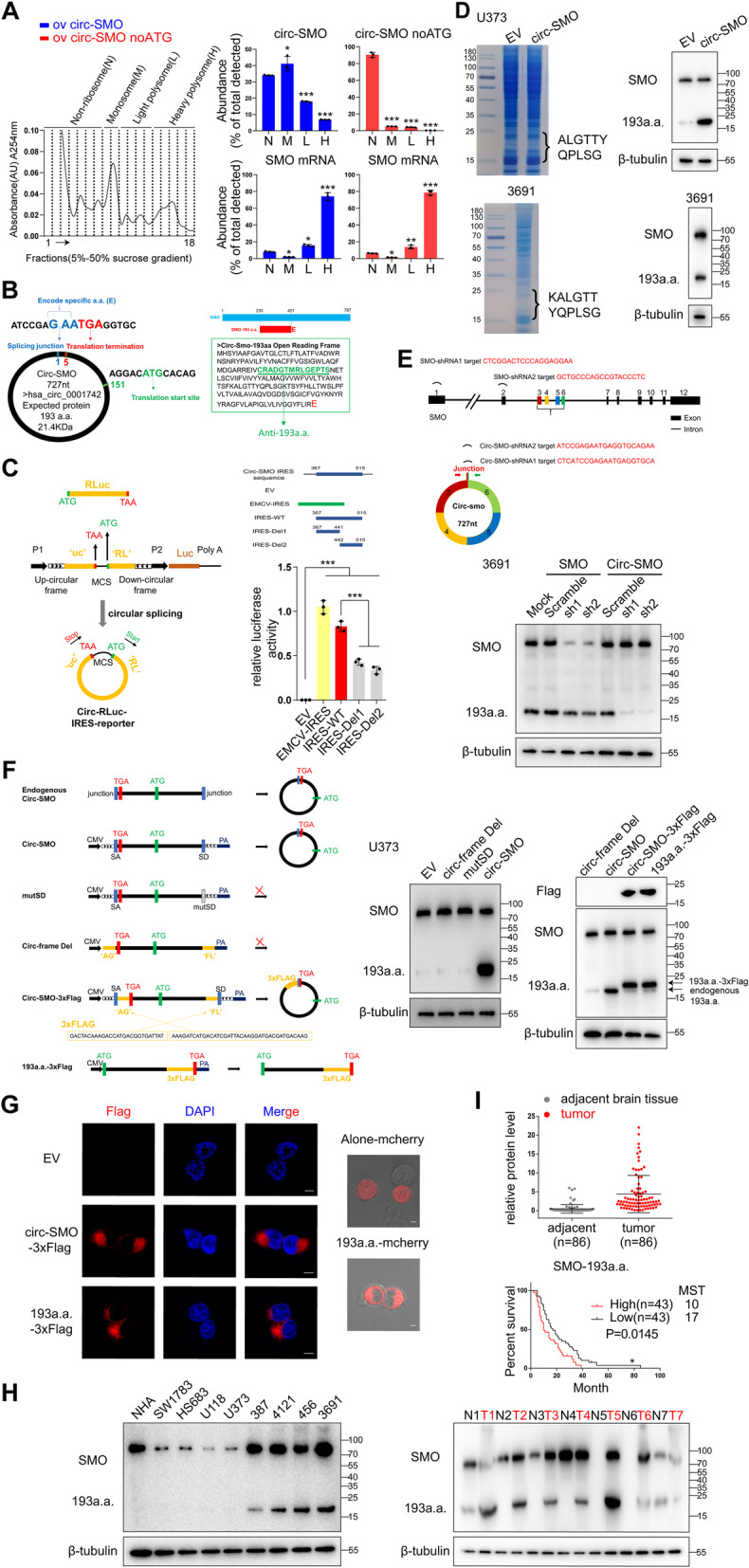
Circ-SMO encodes SMO-193a.a. **a** 293T cells transfected with circ-SMO or circ-SMO noATG plasmid were subjected to polysome profiling assay. Left, Representative polysome profile. Cell lysates were fractionated to collect non-ribosome fractions (N), monosome (M), light polysome (L), and heavy polysome (H) by 5–50% sucrose gradient centrifugation. Dashed lines indicate collected fractions. Right, Detection of circ-SMO and SMO mRNA in indicated fractions by RT-qPCR in 293T cells with indicated modifications. **b** Left, Predicted start and stop codon of ORF in circ-SMO. Right, Predicted amino acid sequences of SMO-193a.a. and antigen sequences for SMO-193a.a. antibody. **c** Identification and activity test of internal ribosomal entry site (IRES) in circ-SMO using circular vector-based luciferase reporter assay. EMCV-IRES was positive control. **d** Immunoblot (IB) and mass spectra (MS) identification of SMO-193a.a. in 373 GBM cells with circ-SMO overexpression (OV) and in 3691 CSC. **e** Upper, Illustration of SMO shRNAs and circ-SMO shRNAs. Lower, SMO and SMO-193a.a. expressions were detected using SMO-193a.a. polyclonal antibody in 3691 CSC treated with indicated shRNAs. **f** Left, Illustration of endogenous circ-SMO, circ-SMO overexpression vector, splicing donor site mutant vector (mutSD), circularization frame deletion vector (circ-frame Del), circ-SMO-3XFlag vector, and linearized SMO-193a.a.-3XFlag vector. Right, IB of cells OV with above described different vectors using anti-SMO-193a.a. antibody or Flag antibody. **g**. Left, Representative immunofluorescence (IF) images of cells OV with indicated vectors and stained with anti-Flag antibody. Bars, 5 μm. Right, Live image of SMO-193a.a.-mCherry in 373 GBM cells. Bars, 5 μm. **h** Left, IB of several established cell lines using anti-SMO-193a.a. antibody. Right, IB of seven random selected GBM samples and adjacent NB using anti-SMO-193a.a. antibody. **i** Upper, semi-quantification of SMO-193a.a. expression based on immunoblot band intensity relative to beta-tubulin in a previously described cohort of 86 GBM samples and paired NB. Lower, Survival analysis was conducted based on SMO-193a.a. expression in 86 GBM patients. The median score of relative expression levels in tumor tissues based on semi-quantification was used as the cutoff to define “high” or “low” SMO-193a.a. expression. MST, median survival time. Lines show the mean ± SD. *p < 0.05, **p < 0.01, ***p < 0.001. Data are representative from at least three experiments with similar results

Protein coding circRNAs were reported recently by our group and others [29, 30]. To assess whether circ-SMO also has coding ability, we first transfected circ-SMO, or circ-SMO with start codon ATG deletion (noATG) into 293T cells. Cell extracts were subjected to 5–50% sucrose gradient centrifugation. Absorbance at 254 nm was measured and fractions were collected. Ribosomes enrichment assay with the non-ribosome fractions (N), monosome (M), light polysome (L), and heavy polysome (H) were shown (Fig. 2a, left). Circ-SMO distribution was then analyzed by qPCR. Circ-SMO was mainly detected in M and L fractions instead of H fractions, which was consistent with a previous report [30]. In contrast, SMO mRNA was mainly localized in H fractions. Deletion of ATG in circ-SMO significantly reduced the ribosomal distribution of circ-SMO but not SMO mRNA, indicating that circ-SMO could be translated (Fig. 2a, right). We further identified an open reading frame (ORF) in circ-SMO, which putatively encoded a 193a.a. novel protein (Fig. 2b, left; Additional file 1: Fig. S1C). We named this protein SMO-193a.a.. SMO-193a.a. shared the same sequence as SMO from amino acid 230 to 421, with an extra Glu in C-terminal (Fig. 2b, right). The ORF in circ-SMO was driven by an internal ribosomal entry site (IRES) sequence (position 367–515), of which the activity was verified by a circular vector-based luciferase reporter assay (Fig. 2c; Additional file 1: Fig. S1D). We also generated an antibody against SMO-193a.a. to validate the active translation of SMO-193a.a. in circ-SMO-OV-transfected 373 cells and in 3691 CSC endogenously. In U373 cells transfected with circ-SMO, SMO-193a.a. expression was identified by immunoblot (IB) and mass spectra (MS) at the predicted molecular weight (Fig. 2d, upper; Additional file 1: Fig. S2A, left; Additional file 3: Table S2). In 3691 CSC, endogenous SMO-193a.a. was also verified by IB and MS (Fig. 2d, lower; Additional file 1: Fig. S2A, right; Additional file 3: Table S2). Notably, this antibody also detected full-length SMO expression, which did not alter by circ-SMO transfection.

CircRNA translation can be driven by N6-methyladenosine (m^6^A) modification [31, 32]. Converse outcome has also been reported that m^6^A modification did not promote exogenous circRNA translation [33], suggested that m^6^A modification could enhance translational efficacies in a part of circRNAs, but may not be required to all translatable circRNAs. In this study, we predicted four m^6^A modification sites on circ-SMO based on circ-SMO sequence analysis [34] (Additional file 1: Fig. S2B). However, in a recently published database based on sequencing data [35], we cannot find any m^6^A modification on circ-SMO (Additional file 1: Fig. S2C). High-throughput sequencing may miss some of the m^6^A sites; m^6^A modification of circ-SMO still needs more experimental validation. In addition, circ-SMO was detected in transcriptome-wide ribosome profiling and polysome profiling data (Additional file 1: Fig. S2C), which further indicated its translational potential.

To exclude the possibility that SMO-193a.a. was translated from an alternative start site inside linear SMO mRNA, linear SMO and circ-SMO shRNAs were used in 3691 CSC (Fig. 2e, upper). Specific knocking down of circ-SMO had no effects on SMO protein level. Knocking down linear SMO (more than 80%) had minor effects on SMO-193a.a. (Fig. 2e, lower). To further assess this possibility, we generated several modified circ-SMO vectors (Fig. 2f, left). Circ-SMO splicing donor site mutant vector (mutSD) and circularization frame deletion vector (circ-frame Del) was used as negative controls. In circ-SMO-3XFlag vector, circularization is required to form the 3XFlag-tag sequences, while linear reading frame in circ-SMO-3XFlag vector could not do the same. Linearized SMO-193a.a.-ORF-3XFlag vector was used as a positive control. Using junction primers-based qPCR, we found transfection of circ-SMO and circ-SMO-3XFlag significantly elevated circ-SMO expression, while transfection of circ-frame-Del and SMO-193a.a.-3XFlag could not (Additional file 1: Fig. S2D). By IB, we found that circ-SMO-3XFlag vector could translate SMO-193a.a. with the 3XFlag tag, as the linearized ORF vector did. Circ-SMO and circ-SMO-3XFlag also enhanced SMO-193a.a. expression. Deletion of the circularization elements in circ-SMO-3XFlag vector or mutation at circ-SMO splicing donor site abolished the SMO-193a.a. expression. (Fig. 2f, right). Together with that we did not observe any disappeared or diminished bands around circ-SMO after RNase R digestion in northern blot (Fig. 1e), these data collectively supported that SMO-193a.a. did not come from linear spliced transcripts of *SMO* gene.

SMO-193a.a. mainly localized in cytoplasmic and cell membrane, as determined by immunofluorescence (IF) and live cell image (Fig. 2g). Furthermore, SMO-193a.a. was enriched in CSCs compared with that in NHA and glioma cell lines (Fig. 2h, left), in GBM samples compared with that in adjacent normal tissues as determined by IB (Fig. 2h, right). Higher SMO-193a.a. predicted worse patients’ total survival in the cohort of 86 GBM patients, as determined by IB semi-quantification-based survival analysis (Fig. 2i). Above results together demonstrated that SMO-193a.a. is encoded by circ-SMO and is a novel oncogenic protein in GBM.

### SMO-193a.a. maintains CSC self-renewal ability

**Fig. 3:**
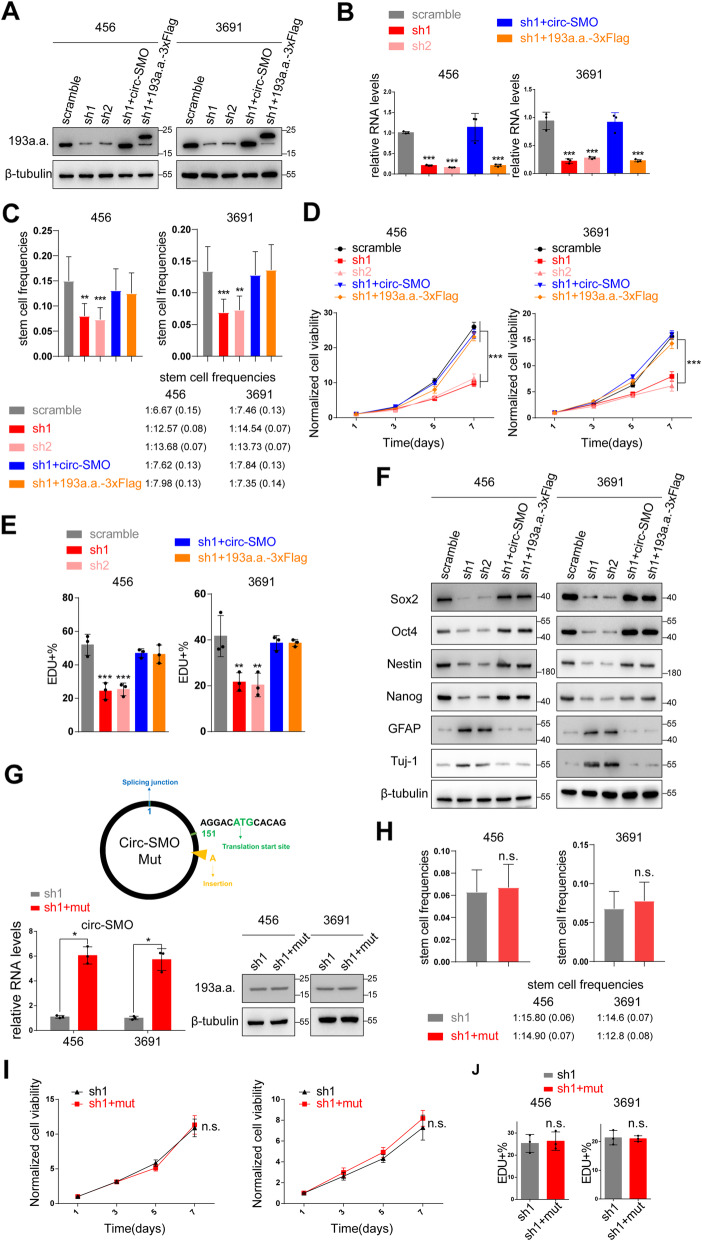
SMO-193a.a. maintains self-renewal of CSCs. **a** IB of SMO-193a.a. in 456, 3691 CSCs stably knocking down (KD) circ-SMO and re-expressed circ-SMO or SMO-193a.a. linearized vector. **b** qPCR of circ-SMO in in 456, 3691 CSCs KD circ-SMO and re-expressed circ-SMO or SMO-193a.a. linearized vector. **c** Limited dilution assay (LDA) of 456, 3691 CSCs with indicated modifications. Stem cell frequencies were calculated. Lines show the estimate values, upper/lower limits of confidence intervals. **d** Cell proliferation of 456, 3691 CSCs with indicated modifications. **e** EdU incorporation assay of 456, 3691 CSCs with indicated modifications. **f** Stemness markers including Sox2, Oct4, Nestin, and Nanog; differentiation markers GFAP and Tuj-1 were determined by IB in 456, 3691 CSCs with indicated modifications. **g** Upper, Illustration of circ-SMO mutant vector. An A was inserted after the start codon of SMO-193a.a. to compromise the ORF. Lower, qPCR and IB were used to verify circ-SMO mutant vector. **h** LDA of 456, 3691 CSCs with indicated modifications. Stem cell frequencies were calculated. Lines show the estimate values, upper/lower limits of confidence intervals. **i** Cell proliferation of 456, 3691 CSCs with indicated modifications. **j** EdU incorporation assay of 456, 3691 CSCs with indicated modifications. Lines show the mean ± SD. *p < 0.05, **p < 0.01, ***p < 0.001. In **a** to **j**, data are representative from at least three experiments with similar results

To investigate SMO-193a.a. function, we established SMO-193a.a. stably knocking down 456 and 3691 CSCs by using two previously described circ-SMO shRNAs (sh1 and sh2) based on their endogenous circ-SMO level. Circ-SMO and SMO-193a.a.-3xFlag plasmid was used to recover SMO-193a.a. expression separately. Sh1 and sh2 successfully inhibited circ-SMO and SMO-193a.a. expression in both CSCs, while circ-SMO and SMO-193a.a.-3xFlag vector could restore SMO-193a.a. expression. Compared with circ-SMO vector, SMO-193a.a.-3xFlag vector did not alter circ-SMO expression in above modified cells (Fig. 3a, b). Functionally, sh1 and sh2 drastically attenuated the sphere formation and sphere sizes in limited dilution assay (LDA) (Fig. 3c; Additional file 1: Fig. S3A, B), inhibited the cellular proliferation (Fig. 3d), and decreased the cell viability (Fig. 3e; Additional file 1: Fig. S3C) in both CSCs. The expression of stemness markers, including Sox-2, Oct4, Nestin, and Nanog, were decreased, while differentiation markers GFAP and Tuj-1 were upregulated (Fig. 3f; Additional file 1: Fig. S3D) after circ-SMO deprivation in both CSCs. Either re-expression of circ-SMO or SMO-193a.a.-3XFlag in above circ-SMO knocking down CSCs recovered the sphere formation, cellular proliferation, and cell viability and promoted the re-expression of stemness markers in CSCs, indicating SMO-193a.a. could generate these biological functions independently (Fig. 3c-f; Additional file 1: Fig. S3A-D). In addition, SMO-193a.a. expression was significantly lower in CD133-negative non-CSC progeny than CD133-positive CSCs (Additional file 1: Fig. S3E). These functional data indicated that SMO-193aa may play an important role in CSC maintenance.

To exclude the possibility that circ-SMO, but not SMO-193a.a., exerted above functions, we constructed a circ-SMO mut plasmid, in which an adenine was inserted after the start codon of SMO-193a.a. ORF to induce a frameshift. The compromised ORF could not translate SMO-193a.a. but should have minimal effect on circ-SMO RNA structure. Re-expression of circ-SMO mut RNA in circ-SMO stable knocking down 456 and 3691 CSCs could not rescue sphere-forming ability, cell proliferation, and EdU incorporation as re-expression of SMO-193a.a. did, further supported that SMO-193a.a. instead of circ-SMO played important functions in maintaining CSC self-renewal and tumorigenicity (Fig. 3g–j; Additional file 1: Fig. S3F).

### SMO-193a.a. activates HH signaling in CSCs

**Fig. 4:**
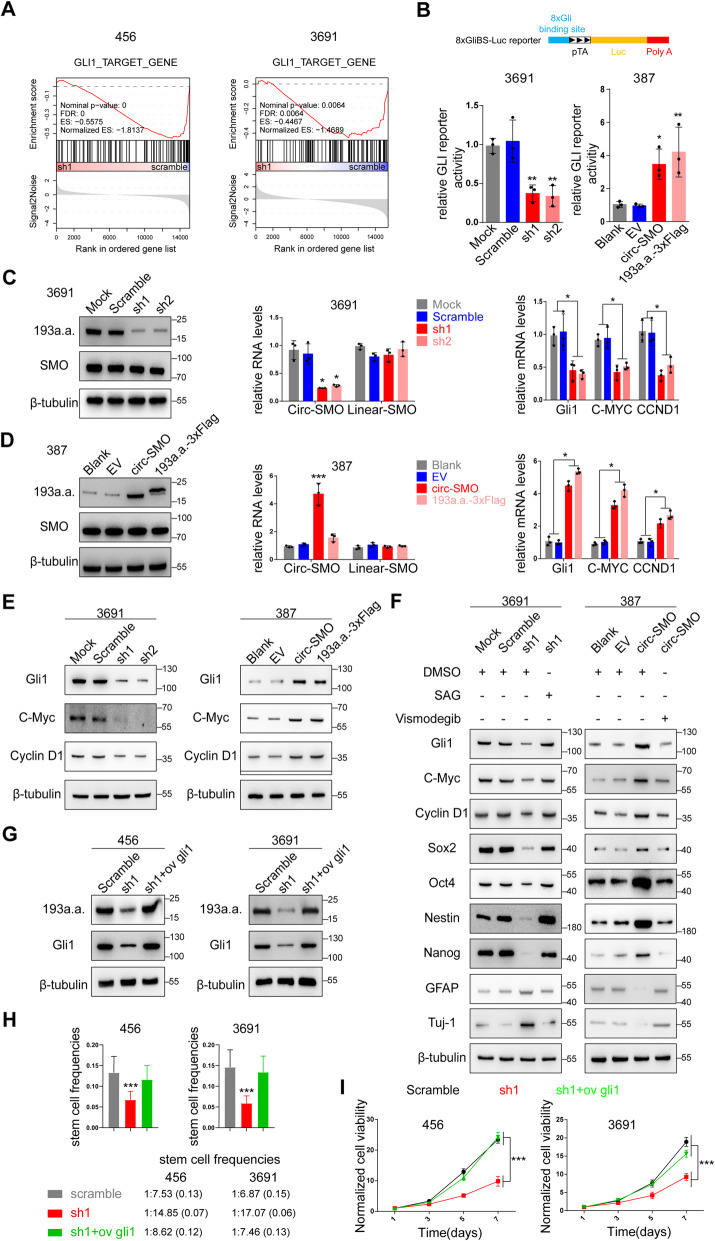
SMO-193a.a. activates HH signaling in CSCs. **a** RNA-seq and Gene Set Enrichment Analysis (GSEA) of 456 and 3691 CSCs treated with circ-SMO scramble shRNA or sh1. **b** Gli1 binding site luciferase assay of 3691 and 387 CSCs with indicated modifications. **c** SMO and SMO-193a.a., SMO mRNA, and circ-SMO, as well as Gli1, C-Myc, and CCND1 mRNA expression were determined by IB or qPCR in 3691 CSC with indicated modifications. **d** SMO and SMO-193a.a., SMO mRNA, and circ-SMO, as well as Gli1, C-Myc, and CCND1 mRNA expression were determined by IB or qPCR in 387 CSC with indicated modifications. **e** IB of Gli1, c-Myc, and cyclin D1 expression in 3691, 387 CSCs with indicated modifications. **f** HH signaling agonist SAG and HH signaling inhibitor vismodegib were used in 3691 CSC with circ-SMO KD or in 387 CSC with circ-SMO OV. IB was used to determine the expression of indicated proteins. **g** Gli1 was OV in 456 and 3691 CSCs with circ-SMO KD. SMO-193a.a. and Gli1 level were determined by IB. **h** LDA was performed in 456 and 3691 CSCs with circ-SMO KD and Gli1 OV. Stem cell frequencies were calculated. Lines show the estimate values, upper/lower limits of confidence intervals. **i** Cell proliferation assay was performed in 456 and 3691 CSCs with circ-SMO KD and Gli1 OV. Lines show the mean ± SD. *p < 0.05, **p < 0.01, ***p < 0.001. In **b** to **i**, data are representative from at least three experiments with similar results

To explore whether Circ-SMO/SMO-193a.a. involve HH signaling in CSCs, we performed RNA-seq and bioinformatic analysis in 456 and 3691 CSCs treated with scramble shRNA or sh1 (Additional file 4: Table S3; Additional file 5: Table S4). Kyoto Encyclopedia of Genes and Genomes (KEGG) enrichment results indicated that circ-SMO/SMO-193a.a. was correlated with pathways directly involving regulation of pluripotency of stem cells, including “Wnt signaling pathway,” “MAPK signaling pathway,” and “PI3K-AKT signaling pathway” (Additional file 1: Fig. S4A). We enrolled a set of HH signaling directly regulated genes determined by Gli1 chromosome immunoprecipitation (ChIP) as previously reported [36]. By using Gene Set Enrichment Analysis (GSEA), we found that genes regulated by Gli1 were also regulated by circ-SMO/SMO-193a.a. in both CSCs with circ-SMO KD (Fig. 4a), which suggested that Gli1 was the downstream target of circ-SMO/SMO-193a.a..

Using a Gli1-Luc reporter, we found that SMO-193a.a. deprivation in 3691 CSC decreased HH signaling activity drastically, while overexpression of SMO-193a.a. in 387 CSC stimulated HH signaling (Fig. 4b). In circ-SMO stably knocking down 3691 CSC, mRNA levels of Gli1, c-Myc, and CCND1, which are all HH signaling downstream targets, were downregulated markedly (Fig. 4c). In contrast, overexpression of circ-SMO or SMO-193a.a.-3XFlag in 387 CSC promoted Gli1, c-Myc, and CCND1 mRNA transcription (Fig. 4d). Importantly, SMO-193a.a. deprivation or overexpression did not affect linear SMO mRNA or protein level, which excluded the possibility that these effects were induced by linear SMO alternation (Fig. 4c, d). SMO-193a.a. expression also positively correlated with protein level of Gli1, c-Myc, and CCND1 in above modified 3691 and 387 CSCs (Fig. 4e). To further testify that SMO-193a.a. exerted its function through HH signaling, we used a SMO agonist SAG [37] in 3691 CSC with stable circ-SMO knocking down. SAG antagonized circ-SMO knocking down induced differentiation, indicated by expression of a series of stemness markers and differentiation markers (Fig. 4f, left). On the other hand, vismodegib abolished circ-SMO overexpression-induced stemness property in 387 CSC (Fig. 4f, right). In 456 and 3691 CSCs with stable circ-SMO knocking down, re-expression of Gli1 recovered the sphere formation frequency in LDA assay and restored the cell proliferation rate (Fig. 4g–i, Additional file 1: Fig. S3G). Interestingly, Gli1 overexpression also increased SMO-193a.a. level (Fig. 4g), suggesting that SMO-193a.a. itself maybe a HH signaling downstream target.

### SMO-193a.a. directly interacts with SMO and promotes SMO activation

**Fig. 5:**
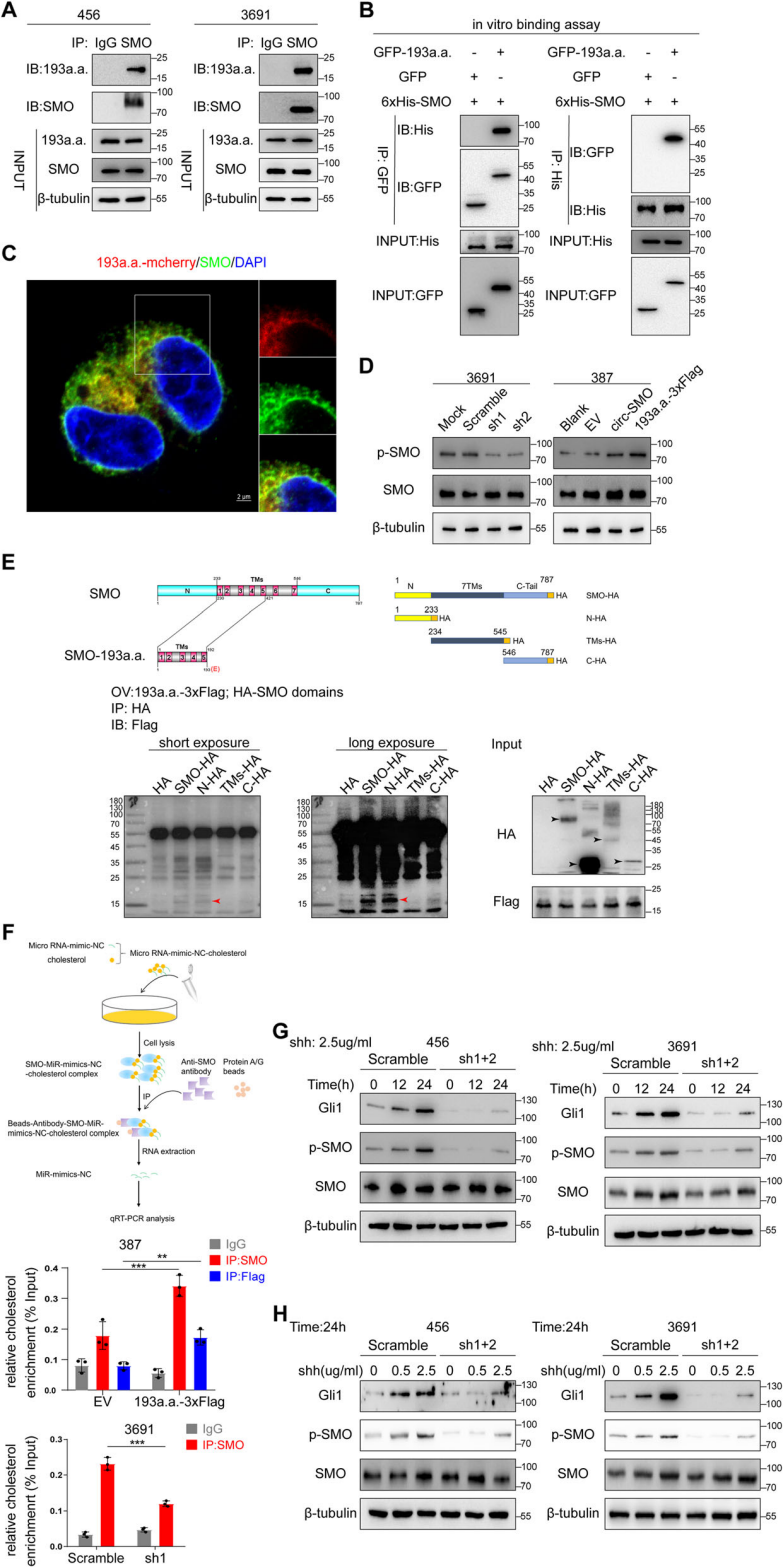
SMO-193a.a. interacts with SMO, promotes SMO activation, and is required for HH-induced SMO de-repression. **a** In vivo IP between SMO and SMO-193a.a. in 456 and 3691 CSCs. **b** In vitro IP using purified SMO and SMO-193a.a.. **c** Colocalization of SMO and SMO-193a.a.-mCherry in 387 CSC. Bars, 2 μm. **d** p-SMO and SMO were determined in 3691 and 387 CSCs with indicated modifications. **e** Upper left, Illustration of SMO-193a.a. 5 transmembrane helix structures and SMO 7 transmembrane helix structures. Upper right, Illustration of full-length and truncated SMO-HA-tagged plasmids. Lower, Full-length and truncated SMO as indicated were co-IP with SMO-193a.a. in 373 GBM cells with SMO-193a.a.-3XFlag and HA-SMO domains OV. **f** Upper, Illustration of cholesterol labeling experiments. A specific nucleotide sequence was labeled with cholesterol and added to 387 CSC with SMO-193a.a. OV or ctrl OV, or 3691 CSC with circ-SMO KD or ctrl KD. Lower, Cholesterol modified SMO or SMO-193a.a. were determined by IP followed qPCR. **g** 2.5μg/ml Shh was added to 456 and 3691 CSCs with indicated modifications. IB was used to determine the expression level of Gli1, p-SMO, and SMO at indicated timepoints. sh1 + 2 defines circ-SMO stable knocking down cells generated by using 1:1 cocktail mixture of sh1 and sh2. **h** Different concentrations of Shh was added to 456 and 3691 CSCs with indicated modifications. IB was used to determine the expression level of Gli1, p-SMO, and SMO after 24 h. Lines show the mean ± SD. *p < 0.05, **p < 0.01, ***p < 0.001. Data are representative from at least three experiments with similar results

Given SMO-193a.a. activated HH signaling without altering SMO protein level, we performed immunoprecipitation (IP) to determine SMO-193a.a.-interacted candidates. We found SMO was a potential binding protein to SMO-193a.a. (Additional file 1: Fig. S4B; Additional file 3: Table S2). In 456 and 3691 CSCs, mutual interaction of SMO and SMO-193a.a. was confirmed by IP (Fig. 5a). Using eukaryotic purified proteins, we confirmed that His-SMO and GFP-SMO-193a.a. directly interacted with each other (Fig. 5b). In SMO-193a.a.-mCherry overexpressed 387 CSC, the colocalization of SMO and SMO-193a.a. was also validated (Fig. 5c). These data demonstrated that SMO-193a.a. regulated SMO activity via direct interaction.

SMO phosphorylation mediated by GRK2 and CKIα was required for HH signaling transduction [38, 39]. As SMO-193a.a. directly interacted with SMO, we next detected phosphorylated SMO level in SMO-193a.a.-modified CSCs. Deprivation of SMO-193a.a. attenuated SMO phosphorylation in 3691 CSC, while SMO-193a.a. overexpression enhanced SMO phosphorylation in 387 CSC (Fig. 5d). These results indicated that SMO-193a.a. enhanced SMO activation. However, SMO-193a.a. alternation did not affect GRK2 or CKIα activity (Additional file 1: Fig. S4C), indicating that SMO-193a.a. controlled SMO activity through an unknown mechanism, which should be in the upstream of GRK2 and CKIα.

### SMO-193aa is required for HH-induced SMO de-repression

Upon HH stimulation, PTCH1 releases SMO from inhibitory status and followed by GRK2 or CKIα phosphorylation. To test whether SMO-193a.a. involved in PTCH1 induced SMO inhibition, we first detected PTCH1 level in SMO-193a.a.-modified 3691 CSC. SMO-193a.a. positively regulated, instead of inhibited, PTCH1 protein level (Additional file 1: Fig. S4D) as PTCH1 was a reported HH signaling downstream target [40]. Furthermore, overexpression of PTCH1 in 387 CSC abolished SMO-193a.a.-induced p-SMO and Gli1 increasement (Additional file 1: Fig. S4D), indicating that PTCH1 could attenuate SMO-193a.a. overexpression-induced SMO activation. These data strongly implied that SMO-193a.a. participated in PTCH1/SMO regulation.

Structural-based study showed that PTCH1 inhibited SMO by reducing inner leaflet cholesterol level [41] and recruitment of cholesterol by CRD domain, which is critical for SMO activation [42]. Notably, a more recent study indicated that the seven transmembrane domains of SMO also bound cholesterol and drove the activation of SMO [5]. SMO-193a.a. shared most of the seven transmembrane domain sequences to form a predicted five transmembrane protein (Fig. 5e, upper; Additional file 1: Fig. S4E). We used several previously reported SMO-truncated constructions [43] and showed that SMO-193a.a. directly bound to the N terminal but not TMs or C-terminal of SMO (Fig. 5e, lower). Based on these results, we hypothesized that SMO-193a.a. may involve in cholesterol translocation to SMO and sequentially control SMO activation. We next synthesized a cholesterol-labeled scramble nucleotide fragment (MiR-mimics-NC-cholesterol) and added it to 387 and 3691 CSCs. The MiR-mimics-NC-cholesterol could attach to SMO-193a.a., if SMO-193a.a. was able to transfer cholesterol to SMO. The amounts of these MiR-mimics-NC-cholesterol on SMO may also change upon SMO-193a.a. modification (Fig. 5f, upper). Using SMO or SMO-193a.a. IP and followed by qPCR targeting these MiR-mimics-NC sequences, we showed that SMO-193a.a. was indeed able to interact with cholesterol and SMO-193a.a. overexpression-increased cholesterol modification of SMO (Fig. 5f, middle). Moreover, MiR-mimics-NC-labeled cholesterol that bound to SMO was reduced markedly in 3691 SMO-193a.a. stable knocking down CSCs compared with that in control cells (Fig. 5f, lower), suggesting that SMO-193a.a. promoted cholesterol modification of SMO.

Next, we determined whether SMO-193a.a. was required for Shh-induced SMO activation. When stimulated SMO-193a.a. stably knocked down 456 and 3691 CSCs with Shh for 24 h, both CSCs exhibited an impaired p-SMO and Gli1 expression, compared with that in scramble shRNA which stably expressed CSCs (Fig. 5g). Similarly, increasing dose of Shh treatment could not stimulate p-SMO and Gli1 expression effectively in SMO-193a.a. knocking down CSCs as that in control CSCs (Fig. 5i). Above data collectively indicated that SMO-193a.a. is required for Shh-induced SMO activation, through enhancing cholesterol modification of SMO.

### SMO-193a.a. is a downstream target of HH signaling

**Fig. 6:**
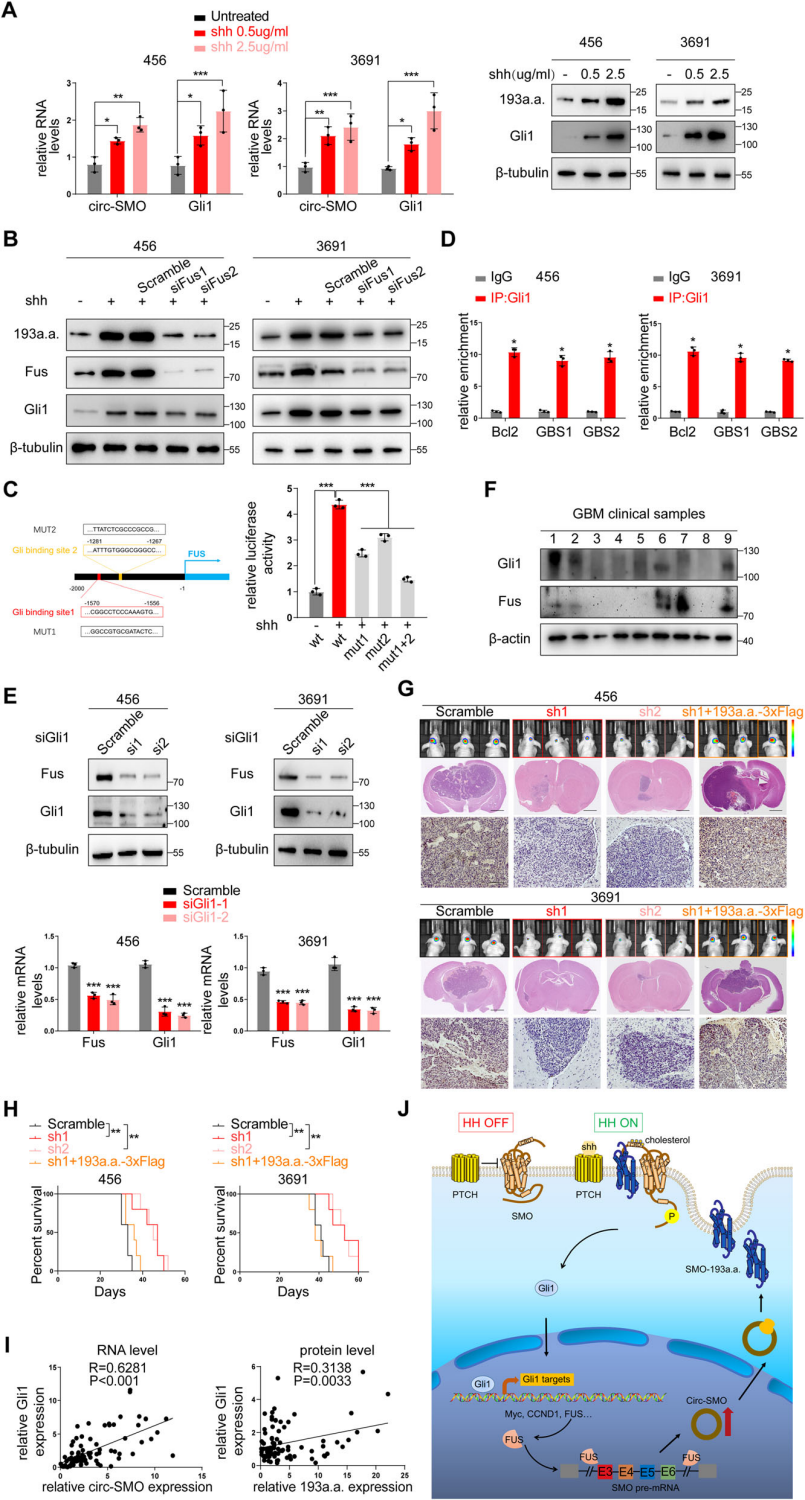
SMO-193a.a. is a downstream target of HH signaling and in vivo effects of SMO-193a.a alteration. **a** Left, circ-SMO expression in 456 and 3691 CSCs treated with Shh. Right, IB of SMO-193a.a. and Gli1 in 456 and 3691 CSCs treated with Shh. **b** IB of SMO-193a.a. and Gli1 in 456 and 3691 CSCs with FUS KD. **c** Luciferase assay of wild type or Gli1 binding site mutated FUS promoter activities after Shh stimulation. **d** ChIP assay of FUS promoter sequences using Gli1 antibody in 456 and 3691 CSCs. Bcl2 was used as positive control. **e** IB and qPCR of FUS protein and mRNA levels in 456 and 3691 CSCs with Gli1 KD. **f** FUS and Gli1 protein level in 9 randomly selected GBM samples. **g** Upper, Representative images of in vivo tumorigenicity assay collected at day 25(456 CSC) and day 30 (3691 CSC) post-implantation using 456 and 3691 CSCs with indicated modifications. Each group contains 5 mice. Lower, Representative images of immunohistochemistry (IHC) of Gli1 expression in above mice. **h** Survival analysis of in vivo tumorigenicity assay using indicated cells. Each group contains 5 mice. **i** Correlation of Gli1 mRNA and circ-SMO in 86 GBM patients and correlation of Gli1 protein and SMO-193a.a. in 86 GBM patients. **j** Graphic abstract. Encoded by circular SMO (circ-SMO), SMO-193a.a. is required for Shh-induced SMO activation, via interacting with SMO, enhancing SMO cholesterol modification and releasing SMO from the inhibition of patched transmembrane receptors 1 (PTCH1). Moreover, circ-SMO/SMO-193a.a. is positively regulated by FUS, a direct transcriptional target of Gli1. Shh/Gli1/FUS/SMO-193a.a. form a positive feedback loop to sustain HH signaling activation in GBM. Lines show the mean ± SD. *p < 0.05, **p < 0.01, ***p < 0.001. Data are representative from at least three experiments with similar results

Gli1 and PTCH1 are transcriptional targets of HH signaling, thereby forming a feedback loop that controls HH signaling intensity and duration upon requirements [11]. Interestingly, circ-SMO and SMO-193a.a. were both dose-dependably upregulated upon increasingly Shh stimulation, suggesting that circ-SMO was also controlled by HH signaling (Fig. 6a). By analyzing side flanking sequences of circ-SMO, we found an RNA-binding protein (RBP), FUS, may promote circ-SMO backsplicing [44] (Additional file 1: Fig. S5A). As expected, knocking down FUS by two specific siRNAs in 456 and 3691 CSCs abolished SMO-193a.a. increasement after Shh stimulation, supporting that FUS positively regulates circ-SMO formation (Fig. 6b). We also identified two Gli1-conservative binding sites in FUS promoter (Additional file 1: Fig. S5B), which implied that FUS was a transcriptional target of HH signaling. After Shh stimulation, FUS promoter’s activity was enhanced significantly. In sharp contrast, mutation of both Gli1 binding sites abolished Shh-induced FUS promoter activation (Fig. 6c). Chromosome IP (ChIP) experiment further indicated that Gli1 could bind to FUS promoter in 456 and 3691 CSCs (Fig. 6d). In both 456 and 3691 CSCs, knocking down Gli1 inhibited FUS mRNA and protein expression (Fig. 6e). Furthermore, FUS expression and Gli1 expression were positively correlated in several randomly selected GBM patient samples from the 86 GBM patient cohort (Fig. 6f). These data indicated that SMO-193a.a. is transcriptionally regulated by FUS, a HH signaling downstream effector.

### In vivo effects of targeting SMO-193a.a in CSCs’ tumorigenicity

Given the critical role of SMO-193a.a. in HH signaling activation, we next tested whether SMO-193a.a. is a potential molecular target for GBM treatment. Stably knocking down circ-SMO in 456 and 3691 CSCs drastically inhibited the in vivo tumorigenicity (Fig. 6g, upper). Restoring SMO-193a.a. expression in the above modified CSCs by SMO-193a.a.-3XFlag promoted the tumor formation (Fig. 6g, upper). Immunohistochemistry (IHC) staining showed that Gli1 was highly expressed in mice xenograft brain tumors, which further demonstrated that SMO-193a.a. enhanced HH signaling activation (Fig. 6g, lower). IHC staining of proliferation (Ki67), CSC (Nestin, Sox2) markers, and TUNEL staining showed the link between SMO-193a.a. and CSCs’ tumorigenicity (Additional file 1: Fig. S5C). Circ-SMO stably knocking down prolonged overall survival of mice model and restoring SMO-193a.a. reversed these survival benefits (Fig. 6h). In the cohort of 86 GBM patients, Gli1 expression was positively correlated with circ-SMO/SMO-193a.a., in both RNA and protein level, which supported the critical role of circ-SMO/SMO-193a.a. in HH signaling activation (Fig. 6i). Previously, we showed that SMO expression was detectable in NHA while SMO-193a.a. was not. In clinical GBM samples and paired NB, SMO-193a.a. was also a better indicator for cancerous tissue than SMO (Fig. 2h). Above data indicated that SMO-193a.a. is a more specific cancerous biomarker than SMO in HH signaling activated GBM and is potentially a novel molecular target for certain GBM patients.

## Discussion

In this study, we identified that exon 3–6 of SMO gene formed a previously undescribed circRNA, circ-SMO. Driven by an active IRES, circ-SMO encoded a novel protein SMO-193a.a.. SMO-193a.a. directly interacts with SMO and enhances SMO cholesterol modification. Moreover, circ-SMO transcription is promoted by FUS, which is a direct downstream target of Gli1, thus Shh/Gli1/FUS/SMO-193a.a. formed a positive feedback loop to sustain HH signaling activation (Fig. 6j). In CSCs and GBM samples, SMO-193a.a. is a more specific biomarker than SMO, which expression is seen in NHA and normal brain tissue. Deprivation of SMO-193a.a. in CSCs reduced the self-renewal and tumorigenesis, which indicated the clinical implication of this newly discovered oncogenic protein.

As a type of newly defined RNA transcript, circRNA’s functions in cancer have been intensively described [20]. Specifically, circRNAs could act as microRNA sponge, protein scaffold, or even template for protein translation, of which all implied the multiple and critical roles circRNAs played in human malignancy, including GBM [19, 20]. CircRNAs are generally lowly expressed in cancer, perhaps due to that the accelerated cellular proliferation rate could affect the RNA splicing process [24]. However, certain circRNAs were also found enriched in tumors, supported by high-throughput sequencing or more specific investigations [45, 46]. A previous report indicated that circ-SMO is ranked top five enriched circRNAs in GBM [47], which was consistent with our findings. The specific expression pattern and higher stability implied that circ-SMO maybe an ideal biomarker for human GBM, while further exploration is clearly warranted in other types of cancers.

We established in this study that circ-SMO encoded SMO-193a.a., which is essential for HH signaling activation in GBM. Structural-based studies had demonstrated some detailed molecular mechanisms of HH signaling transduction. PTCH1 repressed SMO majorly by reducing inner leaflet cholesterol, while Shh-PTCH1 interaction enhanced cholesterol recruitment by SMO [41]. Specifically, CRD domain of SMO is crucial for cholesterol binding, and D95 site modification is required for SMO activation [42, 48]. Meanwhile, a more recent study supported that the transmembrane pocket was also involved in cholesterol binding [5]. Because of sharing with part of the same sequences of transmembrane pocket as SMO, SMO-193a.a. may play a role in interacting and transporting cholesterol. Hu et al. hypothesized that PTCH1 controls the accessibility or enzymatic activity of unknown protein(s) responsible for cholesterol modification of SMO [6]. Based on our results, SMO-193a.a. could be the unraveled protein that transports cholesterol to SMO. Given the critical role of SMO-193a.a., targeting therapy using AAV or small molecular inhibitor is reasonable for next-step investigation, which is currently under way in our lab.

Directly targeting SMO brings inevitable side effects including fetal abnormities, as HH signaling activation is required for embryonic stem cell [4]. Also, acquired resistance to SMO inhibitor is the major cause of treatment failure or recurrence of BCC patients [49]. Compared with SMO, we showed that SMO-193a.a. was more specifically expressed in CSCs and GBM samples. In those SMO-193a.a. overexpressed GBM patients, targeting SMO-193a.a. may avoid side effects from general SMO inhibition or acquired mutation (such as D473, D477 of SMO)-induced SMO inhibitor resistance. Besides, combination inhibition of SMO-193a.a. and SMO, or Gli1 may also provide benefits to those patients who do not respond to single SMO inhibition, such as vismodegib.

## Conclusions

We described a novel oncogenic protein SMO-193a.a., which is encoded by circ-SMO, was essential for HH signaling activation in GBM. SMO-193a.a. de-repressed SMO from PTCH1 upon Shh stimulation via increasing SMO cholesterol modification. Furthermore, circ-SMO is regulated by RNA-binding protein FUS, which is a transcriptional target of Gli1. The Shh/Gli1/FUS/SMO-193a.a. formed a positive loop to sustain constitutive activation of HH signaling in GBM. Our discovery not only describes an unknown mechanism of SMO de-repression during HH signaling activation but also suggests a promising clinical perspective by targeting SMO-193a.a. in HH activated human cancers.

## Methods

### Human cancer and normal tissues

A total of 86 pathologically diagnosed glioma samples and their adjacent normal brain tissues were collected from the Department of Neurosurgery at the 1st Affiliated Hospital of Sun Yat-sen University with written informed consent. The study was approved by the Clinical Research Ethics Committee. All experimental methods comply with the Helsinki Declaration.

### Animal care and ethics statement

Four-week-old female BALB/c-nu mice were purchased from the Laboratory Animal Center of Sun Yat-sen University. The mice were housed in a temperature-controlled (22 °C) and light-controlled specific pathogen-free animal facility with free access to food and water. All experimental protocols concerning the handling of mice were approved by the Institutional Animal Care and Use Committee of Sun Yat-sen University.

### Cell culture and treatments

All cells used in this study were tested for mycoplasma contamination and were authenticated by STR genotyping, in July 2019. The 293T cells were purchased from ATCC (293T ATCC number, CRL-11268). The U373, U118, HS683, and SW1783 cell lines were kindly provided by Dr. Suyun Huang, VCU. Specifically, U373 cells were identical to U251 cells (ECACC 89081403). We used the name of U373 as labeled when it was arrived in our lab. These cells were cultured in Dulbecco’s modified Eagle’s medium (Gibco BRL, Grand Island, NY, USA) supplemented with 10% fetal bovine serum (Gibco BRL, Grand Island, NY, USA) according to standard protocols. NHA were purchased from Lonza and were cultured using an AGM™ Astrocyte Growth Medium Bullet Kit™ (Lonza, Walkersville, MD, USA) as recommended by the manufacturer. Cells above were shifted to 0.5% serum medium and incubated for 24 h before Gli1 and circ-SMO detection. C+ SCs were kindly provided by Dr. Jeremy Rich, UCSD. These cells were cultured in DMEM/F12 medium (Gibco, Grand Island, NY, USA) supplemented with B27 supplement (Life Technologies, Gaithersburg, MD, USA), and bFGF and EGF (20 ng ml^− 1^ each, R&D systems, Minneapolis, MN, USA). Non-CSC progeny was CD133-negative cells derived from CSC using FACS sorting. Human recombinant Shh (GenScript Biotech Corporation, Nanjing, Jiangsu, China) was used with indicated concentrations and time intervals. Vismodegib (50 μM; Beyotime, shanghai, China) or SAG (300 nM; Beyotime, shanghai, China) was added to culture medium for 48 h to inhibit or stimulate SMO activity.

### Antibodies

SMO-193a.a. Rabbit polyclonal antibody was generated by GenScript Biotech Corporation (Nanjing, Jiangsu, China). Antibodies against Sox2 (#ab97959; 1:1000 for IB and IF), Nestin (#ab22035; 1:1000 for IB and 1:200 for IF), GFAP (#ab7260; 1:10000 for IB and 1:1000 for IF), Gli1 (#ab49314; 1:400 for IB, 1:100 for IHC), PTCH1 (#ab53715; 1:500 for IB), GFP-tag (#ab290; 1:1000 for IB and 1μg per 500μg total protein for IP), and Ki67 (#ab15580; 1:500 for IHC) were from Abcam (Cambridge, MA, USA). Antibodies against Gli1(#2643S; 2μg each test for ChIP assay), Oct4 (#2750S; 1:1000 for IB and 1:200 for IF), Nanog (#3580S; 1:1000 for IB and 1:800 for IF), Cyclin D1 (#2978S; 1:1000 for IB), C-Myc (#5605S; 1:1000 for IB), HA-tag (#3724S; 1:1000 for IB and 1μg per 500μg total protein for IP), His-tag (#12698S; 1:1000 for IB), and Sox2 (#3579; 1:400 for IHC) were from Cell Signaling Technology (Danvers, MA, USA). Antibodies against Smo (#sc-166685; 1:100 for IB and 1 μg per 500μg total protein for IP), Fus (#sc-47,711; 1:200 for IB), CK1α (#sc-74582; 1 μg per 500μg total protein for IP), GRK2 (#sc-13143; 1 μg per 500μg total protein for IP), and Nestin (#sc-43927; 1:250 for IHC) were from Santa Cruz Biotechnology, Inc. (Santa Cruz, CA, USA). Anti-Tuj-1 (#MAB1195, 1:1000 for IB and 1:100 for IF) was from R&D Systems (Minneapolis, MN). Anti-Phospho-SMO-S611/615/616 (#AP0940; 1:500 for IB) was from ABclone (Wuhan, Hubei, China). Antibody against Flag (#F1804; 1:1000 for IB, 1 μg per 500μg total protein for IP), beta-Actin (#A1978; 1:5000), and beta-tubulin (#T5201; 1:5000) were from Sigma-Aldrich (St. Louis, MO, USA).

### Plasmids and transfection

Circ-SMO overexpression plasmid, circ-SMO-3xFlag plasmid, 193a.a.-3xFlag plasmid, mutSD plasmid, circ-frame Del plasmid, circ-SMO noATG plasmid, and circ-SMO Mut plasmid were generated by chemical gene synthesis, and pCDH-CMV-MCS-EF1-copGFP-T2A-Puro vector was used as plasmid backbone. For validation of IRES activity, the EMCV-IRES sequence, potential circ-SMO IRES sequence (367-515 bp), and IERS-Delete sequences were chemical synthesized and inserted into the middle of “uc” and “RL” sequences using Circ-RLuc-IRES-Reporter vector. 8xGliBS-Luc plasmid was generated by inserting eight tandem copies of the Gli-binding element (5′-GACCACCCA-3′) into the upstream region of minimal promoter TA and the luciferase gene. For FUS promoter luciferase reporter plasmid construction, the wildtype and mutant FUS promoter region were chemically synthesized and inserted into pGL3-Basic-Luciferase vector (Promega, Madison, WI, USA). SMO-HA, N-HA, TMs-HA, and C-HA vectors use pCDNA3.1(+) plasmid backbone. Plasmids above were obtained from Shanghai Generay Biotech Co, Ltd. (Shanghai, China). PTCH1 and Gli1 overexpression plasmid were obtained from Vigene bioscience, Inc. (Jinan, Shandong, China). The plasmids were transfected with Lipofectamine 3000 (Invitrogen, Carlsbad, CA, USA) according to the manufacturer’s instructions.

### RNA interference (RNAi) and transfection

siRNAs and lentiviral shRNAs were obtained from GenePharma (Suzhou, Jiangsu, China). The target sequences are listed in Additional file 6: Table S5. SiRNA transfection was conducted using Lipofectamine™ RNAiMAX Transfection Reagent (Invitrogen, Carlsbad, CA, USA) as recommended.

### RNA-seq analysis and identification of circRNAs

RNA-seq was performed using an Illumina HiSeqTM 2500. The data were deposited in the SRA database [PRJNA355185 (SRP095744)]. The short reads alignment tool Bowtie2 was used for mapping reads to the ribosome RNA (rRNA) database. The rRNA mapped reads were removed. The remaining reads were further used in alignment and analysis. The removed rRNA reads of each sample were then mapped to a reference genome byTopHat2 (version 2.0.3.12). The reads that could be mapped to the genome were discarded, and the unmapped reads were then collected for circRNA identification. 20mers from both ends of the unmapped reads were extracted and aligned to the reference genome to identify unique anchor positions within the splice site. Anchor reads that aligned in the reverse orientation (head-to-tail) indicated circRNA splicing and were then subjected to find_circ to identify circRNAs. The anchor alignments were then extended such that the complete read aligns and the breakpoints were flanked by GU/AG splice sites. A candidate circRNA was called if it was supported by at least two unique back spliced reads in at least one sample. circRNAs were blasted in the circBase for annotation. Those sequences that could not be annotated were defined as novel circRNAs. CIRIquant software was used for accurate quantification of circRNAs. To identify differentially expressed circRNAs across samples or groups, the edge R package (http://www.r-project.org/) was used. We identified circRNAs with a fold change ≥ 2 and a *P* value < 0.05 in a comparison between samples or groups as significantly differentially expressed circRNAs.

### RNase R treatments

Total RNA was extracted and then treated with RNase R (Lucigen, Middleton, WI, USA) at 37 °C for 15 min according to manufacturer’s instructions. Then RT-qPCR was performed to evaluate the RNase R resistance of circ-SMO.

### Actinomycin D assay

293T cells were equally seeded in 5 wells in 24-well plates (5 × 10^4^ cells per well). Then 24 h later, the cells were treated with actinomycin D (2 μg/ml, HY-17559, MedChem Express, Monmouth Junction, NJ, USA) for 0 h, 4 h, 8 h, 12 h, and 24 h, respectively. After that, the cells were harvested, and the relative RNA levels of circ-SMO and linear SMO were analyzed by qRT-PCR and normalized to the values measured in the 0 h group.

### Northern blotting

Fifteen micrograms total RNA with or without RNase R digestion were separated in a 2% agarose gel using NorthernMax™-Gly Kit from Ambion (Life technologies, Gaithersburg, MD, USA) and transferred to a Hybond-N^+^ membrane (GE Healthcare, Uppsala, Sweden) by capillary transfer. Hybridization was performed with digoxin-labeled oligonucleotide probe specific to exon4 of SMO (exon-probe) or specific to circ-SMO junction (junction probe) (listed in Additional file 6: Table S5). Washing and detection were carried out using DIG Luminescent Detection Kit (Roche, Basel, Switzerland) following the manufacturer’s instructions. After washing, the blots were visualized by expose to X-ray film.

### RNA fluorescence in situ hybridization (FISH)

Cy3-labled oligonucleotide probes complementary to circ-SMO junction region were designed using the Clone Manager suite of analysis tools (Sci Ed Central, listed in Additional file 6: Table S5). In total, 3691 CSCs were seeded on a cover glass-bottom confocal dish and cultured overnight. FISH assay was performed using RNA FISH kit (Suzhou GenePharma Co, Ltd., Suzhou, Jiangsu, China) according to the manufacturer’s instructions. Nuclei were stained with 4,6-diamidino-2-phenylindole (DAPI). Images were acquired on ZEISS LSM 880 with Airyscan (Carl Zeiss Microscopy GmbH, Jena, Germany).

### RNA subcellular isolation

Cytoplasmic and nuclear fractions were isolated using the reagents supplied in RNA subcellular isolation kit (Active Motif, Inc., Carlsbad, CA, USA). Briefly, cells were lysed in complete lysis buffer and incubated for 10 min on ice. After centrifugation, supernatant was transferred for cytoplasmic RNA extraction and the remaining pellet was collected for nuclear RNA purification. RNA products were subjected to qRT-PCR analysis.

### qRT-PCR analysis

PrimeScript™ RT Master Mix (#RR036, Takara, Tokyo, Japan) was used for RNA reverse transcription according to the manufacturer’s instructions if not particularly indicated. Quantitative polymerase chain reaction (qPCR) was performed using TB Green® Premix Ex Taq™ II (Tli RNaseH Plus) (#RR820, Takara, Tokyo, Japan). The primer sequences for genes analyzed are summarized in Additional file 6: Table S5. The relative expression levels were calculated according to 2^−ΔΔCT^.

### Polysome profiling analysis

293T cells were plated in 15-cm plates and transfected with circ-SMO overexpression plasmid or circ-SMO noATG plasmid. After 48 h, the cells were treated with 100 μg/mL cycloheximide in DMSO for 5 min at 37 °C, washed twice with ice-cold 1× PBS containing 100 μg/ml cycloheximide and then harvested by trypsinization for polysome profiling. Cells were lysed in 500 μl polysome lysis buffer (5 mM Tris-HCl (pH 7.5), 2.5 mM MgCl_2_, 1.5 mM KCl, 1× protease inhibitor cocktail (EDTA-free), 0.5% Triton X-100, 2 mM DTT, 0.5% sodium deoxycholate, 100 units RNase inhibitor, and 100 μg/ml cycloheximide] on ice for 15 min, followed by centrifugation at 4 °C for 7 min at 16000×*g* to pellet nuclei and mitochondria. The supernatant was then loaded onto a 5–50%(w/v) sucrose density gradient and ultracentrifuged at 20,000×*g* for 2 h at 4 °C in a Beckman SW41 rotor and subsequently fractionated using BioComp PGFip Piston Gradient Fractionator Model 152. Absorbance at 254 nm was measured using an absorbance detector connected to the fraction collector. RNA was extracted from fractions using TriZol LS solution, and RT-qPCR was conducted to evaluate the Circ-SMO and SMO mRNA levels in indicated fractions.

### Neurosphere formation assay

Neurosphere formation assay was performed by in vitro limiting dilution assay. Briefly, decreasing numbers of cells per well (50, 20, 10, 5, 2, and 1) were plated into 96-well plates. The presence of neurospheres in each well was recorded 7 days after plating. Extreme limiting dilution analysis and stem cell frequency calculation were performed using software available online (http://bioinf.wehi.edu.au/software/elda). All experiments were performed in triplicate.

### Proliferation assay

Cell proliferation experiments were conducted by seeding cells of interest at a density of 1000 cells per well into 96-well plates. At the indicated time points, the cell viability was detected using Cell Counting Kit-8 (Dojindo, Kumamoto, Japan). All data was normalized to day 1 and presented as mean ± SD. All experiments were performed in triplicate.

### EdU incorporation assay

Indicated cells were dissociated with Accutase (Sigma-Aldrich, St. Louis, MO, USA) and seeded on Poly-L-Ornithine (Sigma-Aldrich, St. Louis, MO, USA)-coated coverslips for 24 h. EDU incorporation rates were determined by EdU assay kit (BeyoClick™ EdU Cell Proliferation Kit with Alexa Fluor 594, Beyotime, Shanghai, China) according to the manufacturer’s instruction. All experiments were performed in triplicate.

### Immunofluorescent (IF) staining

Indicated cells were dissociated with Accutase (Sigma-Aldrich, St. Louis, MO, USA) and seeded on Poly-L-Ornithine (Sigma-Aldrich, St. Louis, MO, USA)-coated coverslips for 24 h. Cells were then fixed with 4% paraformaldehyde for 15 min, permeabilized with PBS containing 0.1% Triton X-100 for 5 min at room temperature, blocked with 1% BSA in PBS, and then incubated with primary antibodies overnight at 4 °C followed by appropriate secondary fluorescently labeled antibodies (Invitrogen, Carlsbad, CA, USA) for 1 h at room temperature. Nuclei were counterstained with DAPI. Images were acquired using ZEISS LSM 880 with Airyscan (Carl Zeiss Microscopy GmbH, Jena, Germany).

### Immunoblotting

Briefly, after extraction with RIPA buffer supplemented with protease inhibitor and phosphatase inhibitor cocktails (Pierce Biotechnology, Rockford, IL, USA) and quantified with a BCA kit (Thermo Fisher Scientific, Waltham, MA, USA), equal loading proteins of cell lysates or tissue lysates were denatured by boiling and then resolved by SDS–polyacrylamide gels and then transferred to polyvinylidene fluoride (PVDF) membranes. After blocking with 5% non-fat milk, membranes were successively incubated with indicated primary antibodies and HRP-conjugated secondary antibodies (#31430, #31460, Invitrogen, Carlsbad, CA, USA). The chemiluminescence signals were detected using Clarity™ Western ECL Substrate (Bio-Rad, Hercules, CA, USA). Specifically, for analysis of overexpressed SMO-HA and detection of full-length SMO and SMO-193a.a. in a same blot using SMO-193a.a. Rabbit polyclonal antibody, 2X loading buffer (16% SDS, 100 mM DTT, 2% β-mercaptoehtanol, 0.004% bromophenol blue, 20% glycerol, and100mM Tris-HCl (pH 6.8)) was used and proteins were incubated for 1 h at 37 °C to reduced oligomerization. SDS concentration was upregulated to 24% in 2X loading buffer for TMs-HA detection. Standard loading buffer and boiling procedure was used if not specifically indicated.

### LC-MS analysis

Proteins were separated via SDS-PAGE and subjected to digestion with sequencing-grade trypsin (Promega, Madison, WI, USA). The digested peptides were analyzed with a QExactive mass spectrometer (Thermo Fisher Scientific, Waltham, MA, USA). The fragment spectra were analyzed using the National Center for Biotechnology Information nonredundant protein database with Mascot (Matrix Science, Boston, MA, USA).

### Immunoprecipitation (IP)

Cells were lysed in co-IP soft RIPA Lysis Buffer (#P0013D, Beyotime, Shanghai, China) supplemented with protease and phosphatase inhibitors. The supernatant was collected and subjected to immunoprecipitation using indicated primary antibodies at 4 °C overnight. Then the lysates were incubated with 30 μl protein A/G agarose (Gibco BRL, Grand Island, NY, USA) for 2 h at room temperature. The collected agarose-protein complexes were centrifugated and washed with cold PBST (PBS containing 0.1% Tween20) for 5 times and then subjected to SDS-PAGE and analyzed by LC-MS or Western blotting.

### In vitro binding assay

293T cells were transfected separately with GFP-tagged and 6xHis-tagged proteins. GFP-193a.a. and GFP were purified using anti-GFP antibody (#ab290, Abcam) and Catch and Release® v2.0 Reversible Immunoprecipitation System (Millipore, Burlington, MA, USA); 6xHis-SMO were purified with HisPur™ Ni-NTA Purification Kit (Thermo Fisher Scientific, Waltham, MA, USA). For in vivo binding assay, purified GFP or GFP-193a.a. were incubated with 6xHis-SMO for 4 h at 4 °C and then subjected to immunoprecipitation with the indicated primary antibodies at 4 °C overnight. Then, the protein complexes were collected by incubation with 30 μL protein A/G agarose (Gibco BRL, Grand Island, NY, USA) for 2 h at room temperature, followed by washing with cold PBST buffer 5 times and then subjected to western blotting.

### IP-Kinase assay

Cells were lysed in co-IP buffer, and non-denatured CK1α and GRK2 proteins for kinase assay were obtained using Catch and Release® v2.0 Reversible Immunoprecipitation System (Millipore, Burlington, MA, USA) according to the manufacturer’s instructions. In brief, 500 μg of indicated cell lysates were incubated with anti-CK1α antibody (#sc-74582, Santa Cruz Biotechnology, Inc) or anti-GRK2 antibody (#sc-13143, Santa Cruz Biotechnology, Inc) and 10 μl of antibody capture affinity ligand in a Catch and Release v2.0 spin column. After 12 h end-over-end shaking, the column was centrifuged, washed, and then eluted with non-denaturing elution buffer. The IP-CK1α and IP-GRK2 eluates were subjected to further kinase assay using CK1α1 Kinase Enzyme System and GRK5 Kinase Enzyme System (Promega, Madison, WI, USA) respectively according to the manufacturer’s instructions. Briefly, indicated eluates were incubated with ATP/substrate Mix for 60 min at room temperature, followed by ADP detection with ADP-Glo™ Kinase Assay (Promega Madison, WI, USA).

### MiR-mimics-NC-cholesterol binding and detection assay

Micro RNA-mimics-NC was synthesized with 5′ cholesterol modification from GenePharma (Suzhou, Jiangsu, China). Under cholesterol depletion condition (serum depletion medium with 1 μM lovastatin and 10 μM mevalonate), 2 μg/ml MiR-mimics-NC-cholesterol was applied to maximally replace cholesterol. After 16 h, cells were harvested and lysed with co-IP soft RIPA Lysis Buffer supplemented with protease and phosphatase inhibitors and RNasin® Ribonuclease Inhibitor (1:400, Promega, Madison, WI, USA), and then equal amount of total protein was subjected to immunoprecipitation using SMO antibodies at 4 °C overnight. Then the lysates were incubated with 30 μl protein A/G agarose for 2 h at room temperature. The collected agarose-protein complexes were centrifugated and washed with cold 0.1% DEPC pre-treated PBST (PBS containing 0.1% Tween20) for 5 times. The precipitates were subjected to RNA extraction using TRIzol™ Reagent (Invitrogen, Carlsbad, CA, USA), followed by qRT-PCR analysis using equal volume of total RNA. Reverse transcription primer and PCR primer were listed in Additional file 6: Table S5. The relative expression levels were calculated according to 2^−ΔΔCT^. MiR-mimics-NC enrichment of sample manifested the amount of cholesterol binding to SMO.

### IRES activity validation and Gli-Luciferase reporter assay

293T cells were transfected with empty Circ-RLuc-IRES-Reporter vector, EMCV-IRES vector, IRES wildtype, or deletion vectors and incubated for 48 h for analyzing putative IRES activity. For Gli-luciferase reporter assay, indicated cells were seeded in six-well plates and transfected with 8xGliBS-Luc plasmid combined with pRL-TK vector (10:1 ratio) as an internal control. After 48 h, the cells were rinsed with PBS and subjected to dual luciferase assay. A dual luciferase reporter assay system (Promega, Madison, WI, USA) was used based on the manufacturer’s instructions. Firefly luciferase activity was normalized to Renilla luciferase activity for each sample. Data were from three independent assays.

### FUS promoter luciferase reporter assay

Series of pGL3 reporter plasmids carrying wildtype or mutant promoter region of FUS and pRL-TK vectors were transfected into 293T cells. After 24 h, cells were stimulated with Shh (2.5 μg/ml) for 24 h, followed by luciferase activity analysis using a dual luciferase reporter assay system (Promega, Madison, WI, USA). The promoter activity of constructed plasmid was normalized with Renilla luciferase activity. Experiments were performed in triplicate.

### ChIP-PCR assay

ChIP assays were performed using Simple ChIP Plus Enzymatic Chromatin IP Kits (#9003; Cell signaling Technology, Danvers, MA, USA) according to the manufacturer’s instructions. Cell lysates were incubated with 2 μg of anti-Gli1 antibody (#2643S; Cell Signaling Technology) or rabbit IgG. The resultant DNA was subjected to qPCR for further analysis. Primers were listed in Additional file 6: Table S5.

### In vivo tumorigenicity assay

All mouse experiments were approved by the Institutional Animal Care and Use Committee of the Sun Yat-sen University. We intracranially implanted 2000 indicated cells into 4-week-old female athymic nude mice (purchased from the Animal center, Sun Yat-sen University). Five mice were injected for each group. For in vivo bioluminescence imaging, all 456 and 3691 cells were transduced with firefly luciferase through lentiviral infection prior to other transfection. Mice were anesthetized with isoflurane and injected intraperitoneally with 120 mg/kg body weight luciferin solution (VivoGlo™ luciferin, Promega, Madison, WI, USA). Images were acquired with the Xenogen IVIS Lumina series II (Xenogen Corporation, Alameda, CA, USA). Mice were sacrificed at indicated time points and their brains were harvested, fixed in 4% formaldehyde, embedded in paraffin, and then subjected to hematoxylin and eosin staining and IHC staining. For the survival experiments, mice were monitored until they developed neurologic symptoms that significantly inhibited their life qualities (such as seizures, ataxia and lethargy, and inability to feed) or 100 days post-implantation. The overall survival curves were calculated with the Kaplan−Meier method and compared by the Log-rank test.

### Immunohistochemistry (IHC) and TUNEL staining

Paraffin-embedded brain tissues were sectioned at 4-mm thickness. Xylene and ethanol of sequential concentrations was used for dewax and hydration. Antigen retrieval was performed using microwave for 20 min in 0.01 M citrate buffer (pH 6.0), followed by cooling to room temperature. After blocking by 3% H_2_0_2_ and then 10% FBS, samples were incubated with anti-Gli1 antibody (1:100; #ab49314; Abcam), anti-Ki67 antibody (1:500; #ab15580; Abcam), anti-Nestin antibody (1:250, #sc-43927, Santa Cruz), and anti-Sox2 antibody (1:400; #3579; CST) overnight at 4 °C and secondary antibody 30 min at room temperature. TUNEL staining was conducted using Colorimetric TUNEL Apoptosis Assay Kit (#C1098; Beyotime) based on the manufacturer’s instructions. Immunodetection was performed using DAB solution. Tissues were counterstained with hematoxylin.

### Statistical analysis

Statistical tests were conducted using GraphPad Prism (Version 8; La Jolla, CA, USA) software unless otherwise indicated. The data are presented as the mean ± standard deviation (S.D.) from three independent experiments. For the comparison of parametric data between glioma samples and their adjacent normal brain tissues, paired, two-tailed Student’s *t* tests were used. For other parametric data, unpaired, two-tailed Student’s *t* tests or one-way ANOVA were used. OS curves were assessed with the Kaplan−Meier method and compared by the Log-rank test. The correlations were calculated by Pearson correlation analysis. Data distribution was assumed to be normal, but this was not formally tested. A level of *P* < 0.05 was used as the cutoff for significant differences. For all experiments, analyses were done in biological triplicates. No animals or data points were excluded from the analyses for any reason. Blinding and randomization were performed in all experiments. Statistical analyses for the RNA-seq data are described in the respective sections.

## Supplementary Information

**Additional file 1:** **Fig. S1–5** with figure legends.

**Additional file 2:** **Table S1.** Differential expressed circRNAs in GBM compared with that in NB by CIRIquant analysis.

**Additional file 3:** **Table S2.** The mass spectrometry analysis data.

**Additional file 4:** **Table S3.** Differential expressed genes and KEGG enrichment analysis in 456 CSC treated with scramble shRNA or sh1.

**Additional file 5:** **Table S4.** Differential expressed genes and KEGG enrichment analysis in 3691 CSC treated with scramble shRNA or sh1.

**Additional file 6:** **Table S5.** Primers and oligoes used in this paper.

**Additional file 7:** Uncropped Northern and Western blots.

**Additional file 8:** Review history.

## Data Availability

The RNA-seq data of glioblastoma tissue (PRJNA525736) and 456 and 3691 CSCs (PRJNA657303) in this study were deposited in the SRA database (https://www.ncbi.nlm.nih.gov/sra). The mass spectra data were provided in Additional file 3: Table S2. For all other materials request, please contact the corresponding author at zhangnu2@mail.sysu.edu.cn.
