# Supplementary figures and images for: A novel protein encoded by circular SMO RNA is essential for Hedgehog signaling activation and glioblastoma tumorigenicity
Source: Genome Biol. 2021 Jan 14;22:33. doi: 10.1186/s13059-020-02250-6 (PMC7807754; doi:10.1186/s13059-020-02250-6)

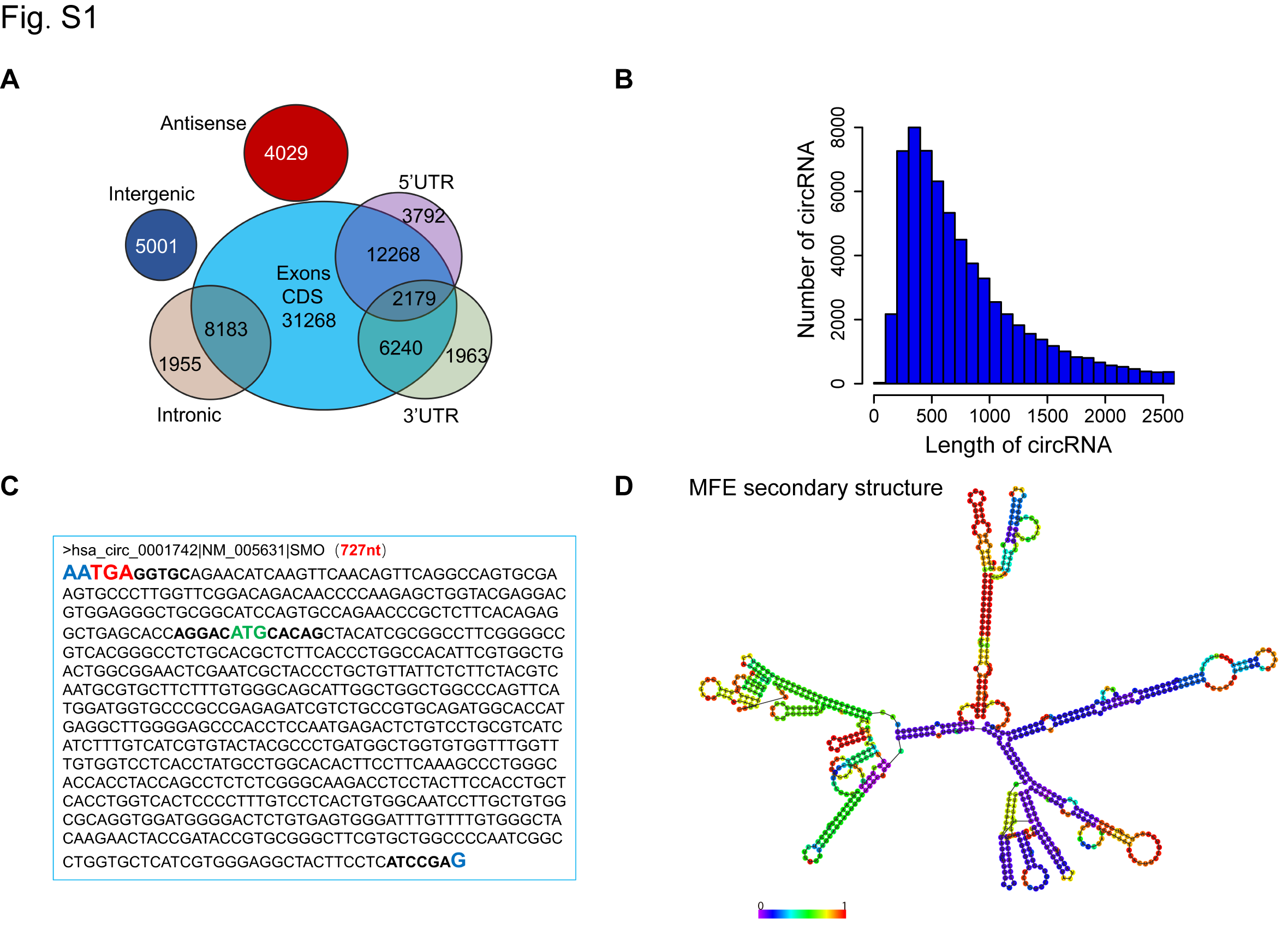

Supplement: Supplementary file 1 — Additional file 1: Fig. S1–5 with figure legends. [file 13059_2020_2250_MOESM1_ESM.zip › Fig. S1.tif]

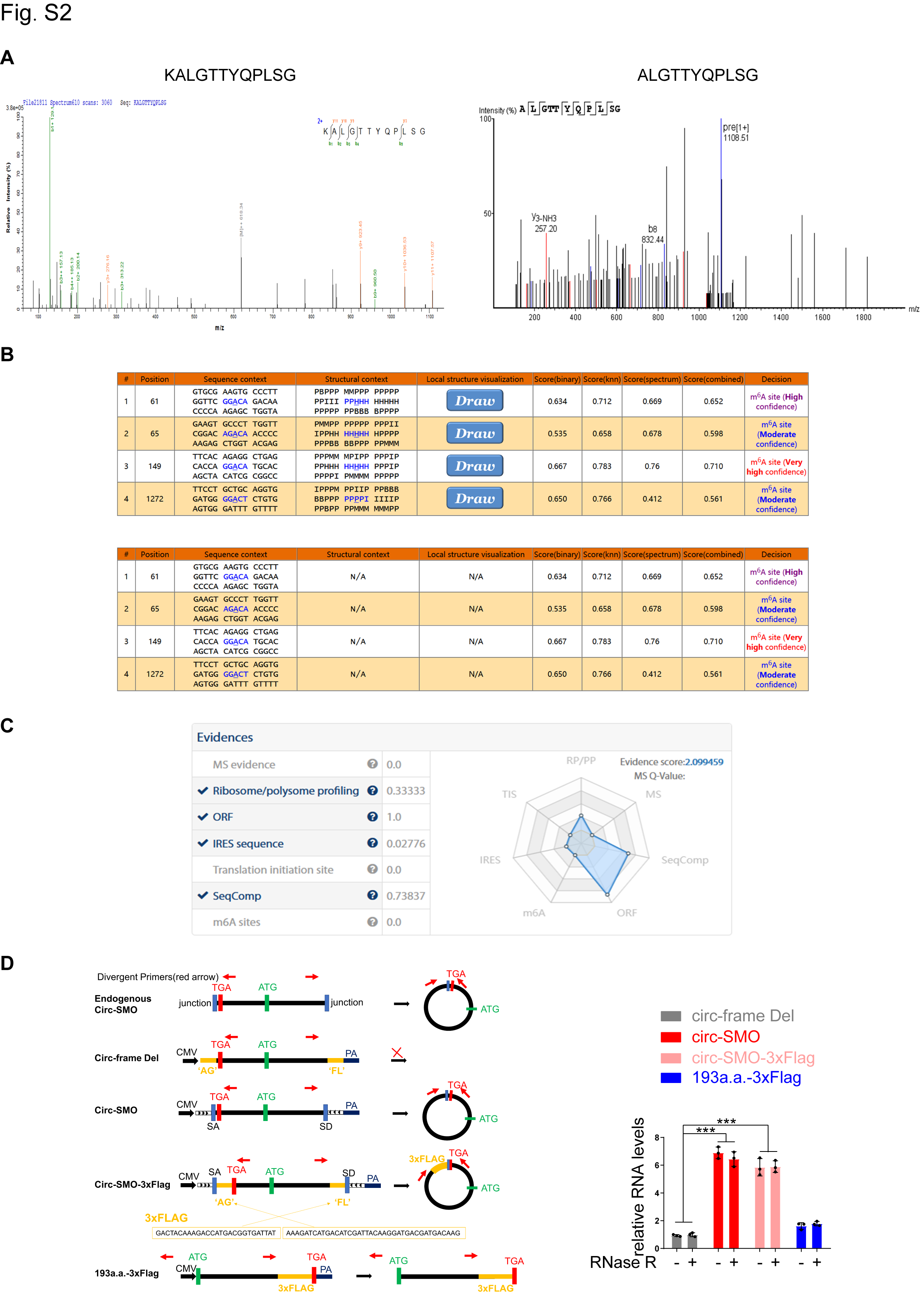

Supplement: Supplementary file 1 — Additional file 1: Fig. S1–5 with figure legends. [file 13059_2020_2250_MOESM1_ESM.zip › Fig. S2.tif]

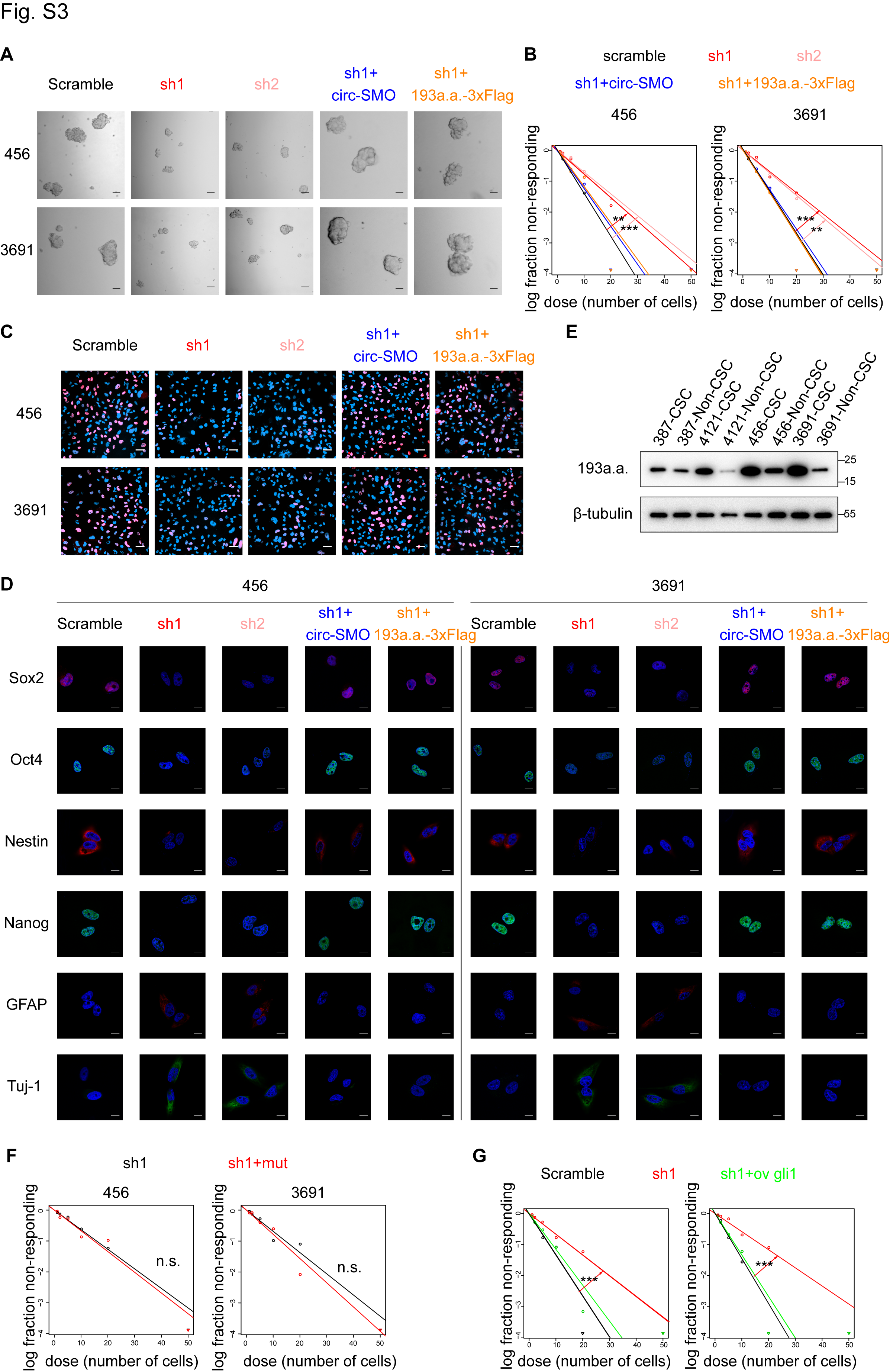

Supplement: Supplementary file 1 — Additional file 1: Fig. S1–5 with figure legends. [file 13059_2020_2250_MOESM1_ESM.zip › Fig. S3.tif]

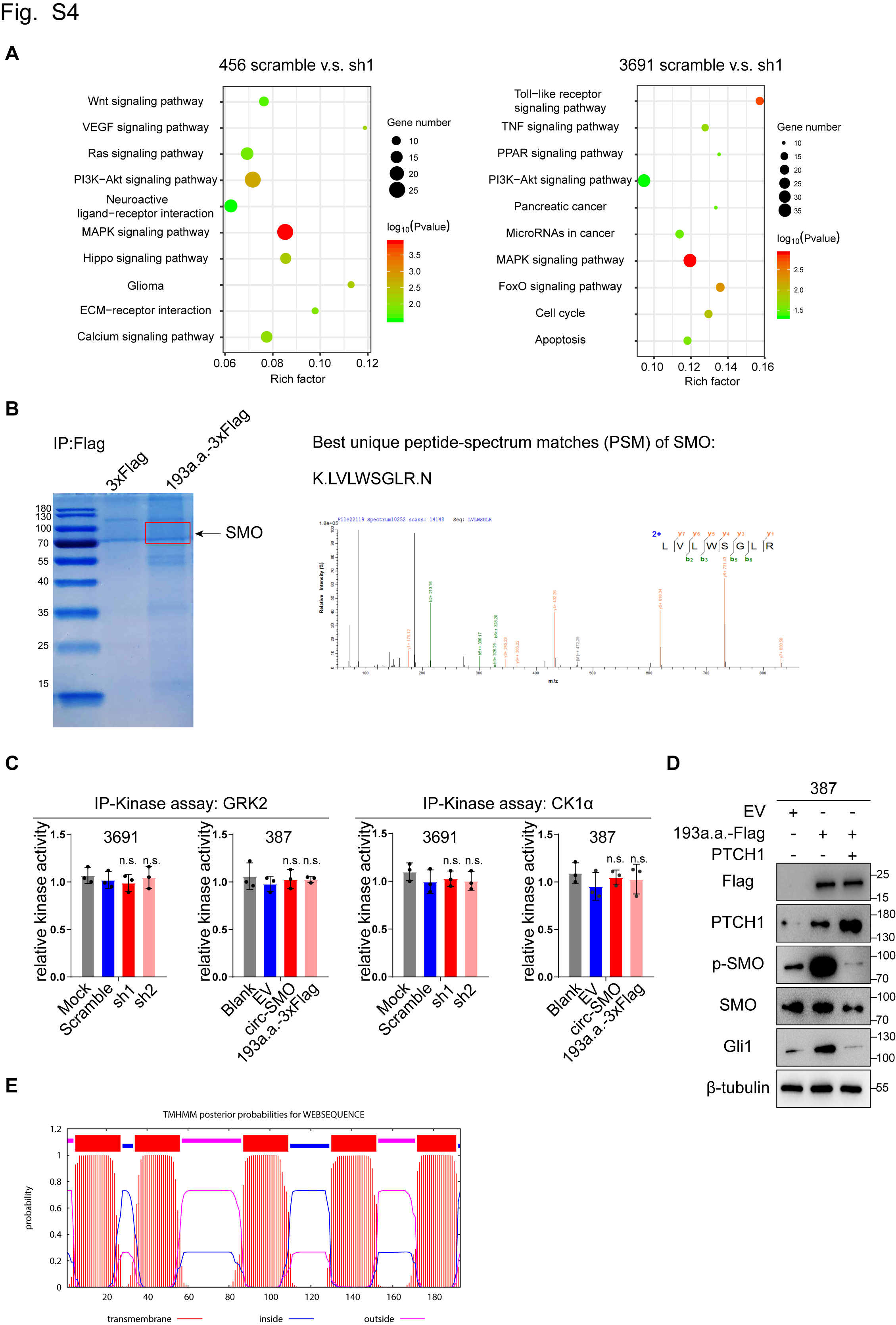

Supplement: Supplementary file 1 — Additional file 1: Fig. S1–5 with figure legends. [file 13059_2020_2250_MOESM1_ESM.zip › Fig. S4.tif]

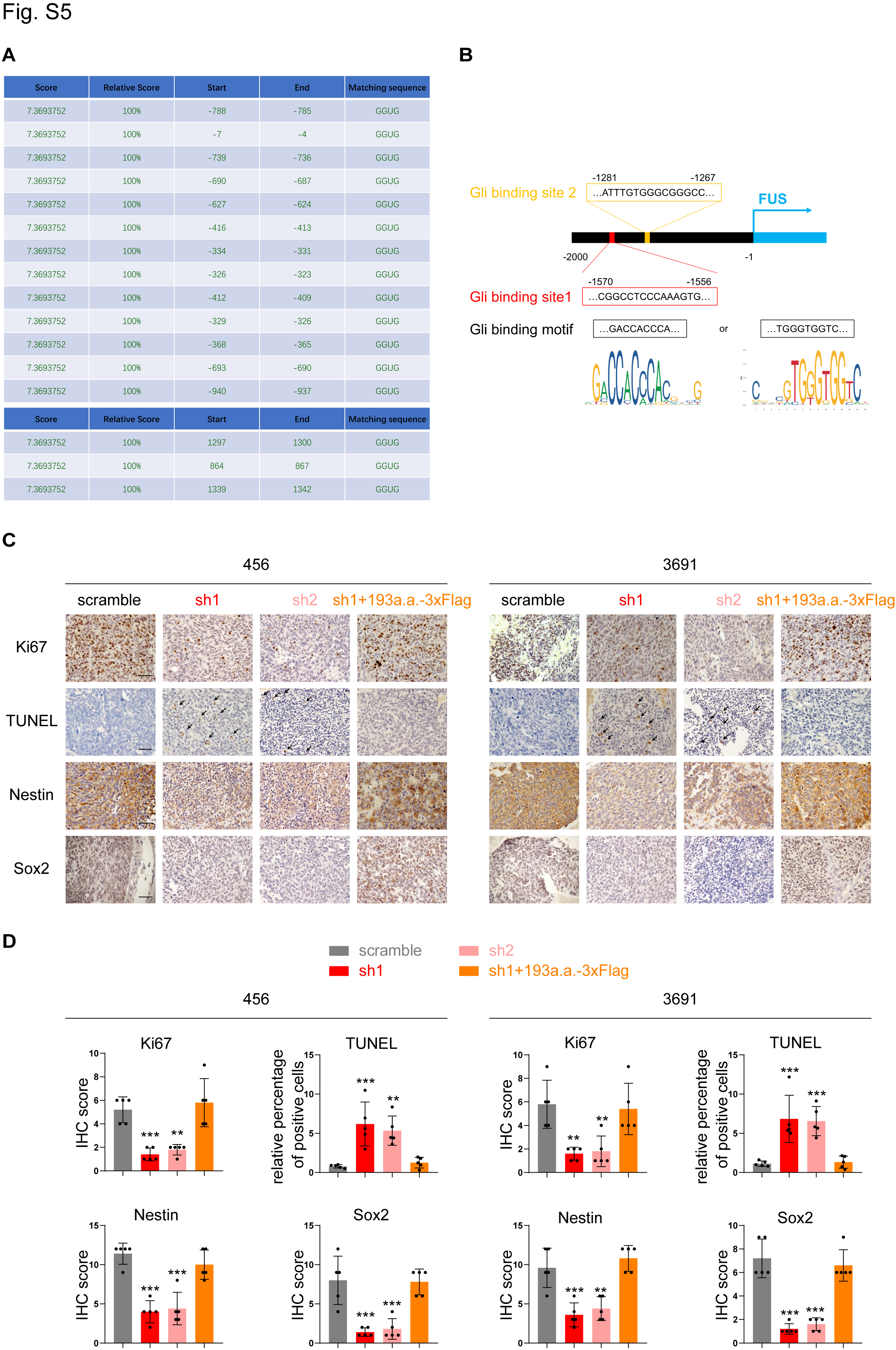

Supplement: Supplementary file 1 — Additional file 1: Fig. S1–5 with figure legends. [file 13059_2020_2250_MOESM1_ESM.zip › Fig. S5.tif]
