# Supplementary material for: A novel protein encoded by circular SMO RNA is essential for Hedgehog signaling activation and glioblastoma tumorigenicity
Source: Genome Biol. 2021 Jan 14;22:33. doi: 10.1186/s13059-020-02250-6 (PMC7807754; doi:10.1186/s13059-020-02250-6)
Supplement: Supplementary file 6 — Additional file 6: Table S5. Primers and oligoes used in this paper. [file 13059_2020_2250_MOESM6_ESM.docx]

| qRT-PCR Primers | Forward primer (5‘to 3’) | Reverse primer (5‘to 3’) |
| --- | --- | --- |
| Circ-SMO (divergent primer) | TCCTCACTGTGGCAATCCTT | TGATGTTCTGCACCTCATTCTC |
| Linear-SMO (convergent primer) | AGGCCAGGAGCTGTCCTTCA | ACAGGTTGGCTTGTTCCTCT |
| Circ-SMO and Circ-SMO-3xFlag (divergent primer2) | GGCTACTTCCTCATCCGAGAAT | CGTCCTCGTACCAGCTCTTG |
| β-Actin | ACAGAGCCTCGCCTTTGCCGAT | CTTGCACATGCCGGAGCCGTT |
| U6 | GTGCTCGCTTCGGCAGCACAT | ATGGAACGCTTCACGAATTTG |
| Gli1 | CAGTACATGCTGGTGGTTCA | TGGCTCGGTCACTGGCATTG |
| C-MYC | CAGCTGCTTAGACGCTGGAT | CGAGTCGTAGTCGAGGTCAT |
| CCND1 | GCTGCGAAGTGGAAACCATC | CCTCCTTCTGCACACATTTGAA |
| FUS | GCCAAGATCAATCCTCCATGAGTAGTG | TCCACGGTCCTGCTGTCCATAG |
| MiR-mimics-NC-cholesterol RT primer | GTCGTATCCAGTGCAGGGTCCGAGGTAT  TCGCACTGGATACGACGTCACGT |  |
| MiR-mimics-NC-cholesterol | TTACGTTCTCCGAACGTGTCA | AGTGCAGGGTCCGAGGTAT |
| ChIP-PCR Primers | **Forward primer (5‘to 3’)** | **Reverse primer (5‘to 3’)** |
| Bcl2 | ACACACGTCTGCGAGTGTGAATGT | TCCCTCTGTCCCTAACACCTTT |
| GBS1 | AACTCCTGACCTCGTGATCC | GTATGTTCCAGGCACTGTTC |
| GBS2 | GCTCTCACTGGAGAGTTGAA | TTCAGAAGGTCCTTTGAGGGTT |
| shRNA targets | **Targets** |  |
| Circ-SMO shRNA1 | CTCATCCGAGAATGAGGTGCA |  |
| Circ-SMO shRNA1 | ATCCGAGAATGAGGTGCAGAA |  |
| Scramble shRNA | GTGAACGGTGTAAGTGCTAGT |  |
| SMO shRNA1 | CTCGGACTCCCAGGAGGAA |  |
| SMO shRNA2 | GCTGCCCAGCCGTACCCTC |  |
| Scramble shRNA | TTCTCCGAACGTGTCACGT |  |
| siRNA sequences | **Sense (5‘to 3’)** | **Antisense (5‘to 3’)** |
| FUS–siRNA1 | GUGGAGGUGGAGGUAACUAtt | UAGUUACCUCCACCUCCACtt |
| FUS–siRNA2 | UGGAGGUGGAGGUAACUAUtt | UAGUUACCUCCACCUCCAtt |
| Gli1-siRNA1 | GCCACCAAGCUAACCUCAUtt | AUGAGGUUAGCUUGGUGGCtt |
| Gli1-siRNA2 | GCCUGAAUCUGUGUAUGAAtt | UUCAUACACAGAUUCAGGCtt |
| scramble siRNA | UUCUCCGAACGUGUCACGUtt | ACGUGACACGUUCGGAGAAtt |
| MiR-mimics-NC-cholesterol | **5‘to 3’** |  |
|  | Cholesterol-UUCUCCGAACGUGUCACGUGAC |  |
| Probe | **5‘to 3’** |  |
| FISH probe | Cy3-TTCTGCACCTCATTCTCGGATGAGGAA |  |
| Northern blotting exon-probe | CACGGCAGACGATCTCTCGGCGGGCACCATCCAT  GAACTGGGCCAGCCAG**-DIG** |  |
| Northern blotting junction-probe | TTGAACTTGATGTTCTGCACCTCATTCTCGGATGA  GGAAGTAGCCTCCCACG-**DIG** |  |
