# Supplementary material for: A novel protein encoded by circular SMO RNA is essential for Hedgehog signaling activation and glioblastoma tumorigenicity
Source: Genome Biol. 2021 Jan 14;22:33. doi: 10.1186/s13059-020-02250-6 (PMC7807754; doi:10.1186/s13059-020-02250-6)

Uncropped Northern blots for Figure 1

E

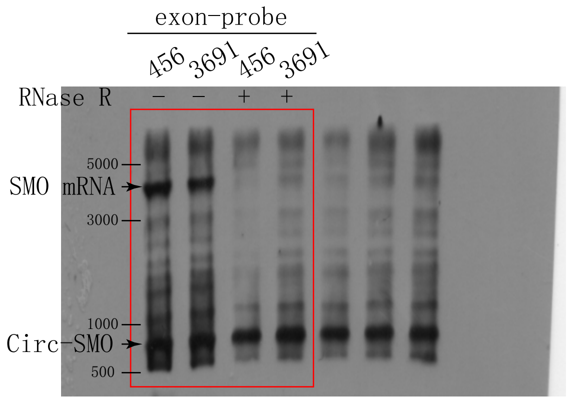

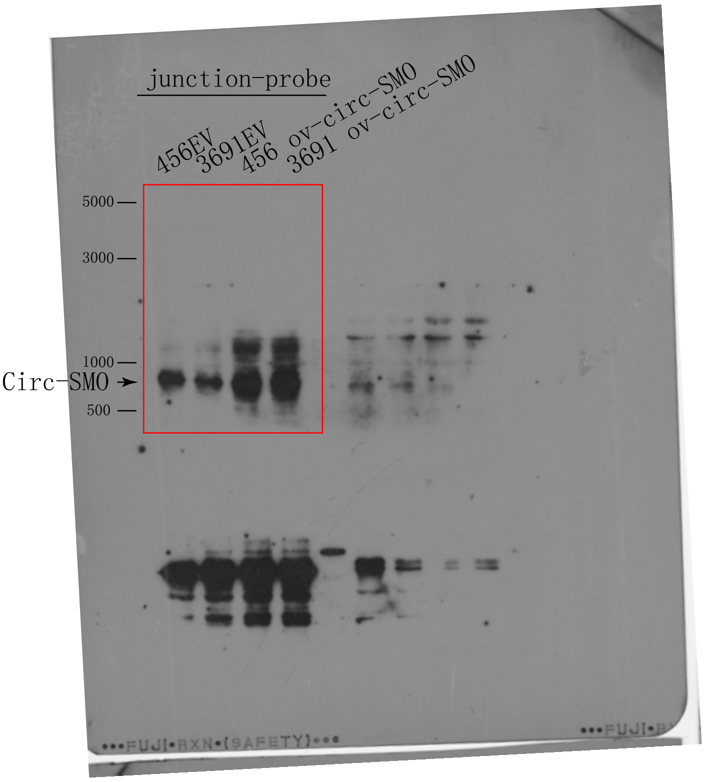

Uncropped Western blots for Figure 2

D

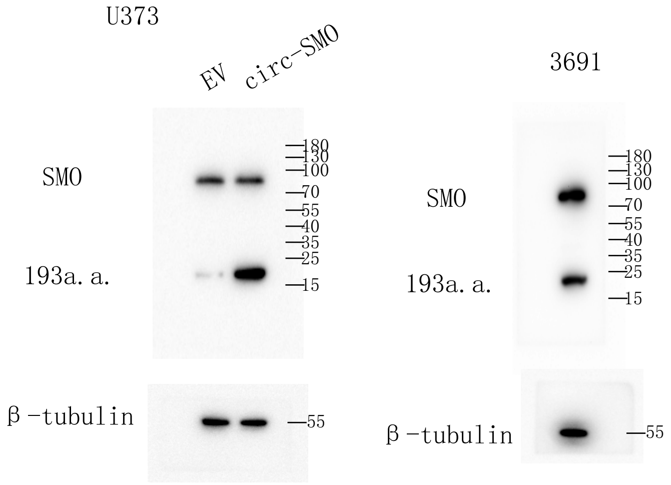

E

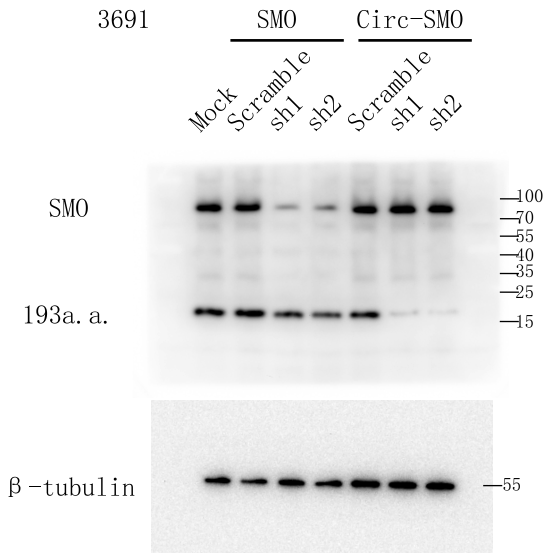

F

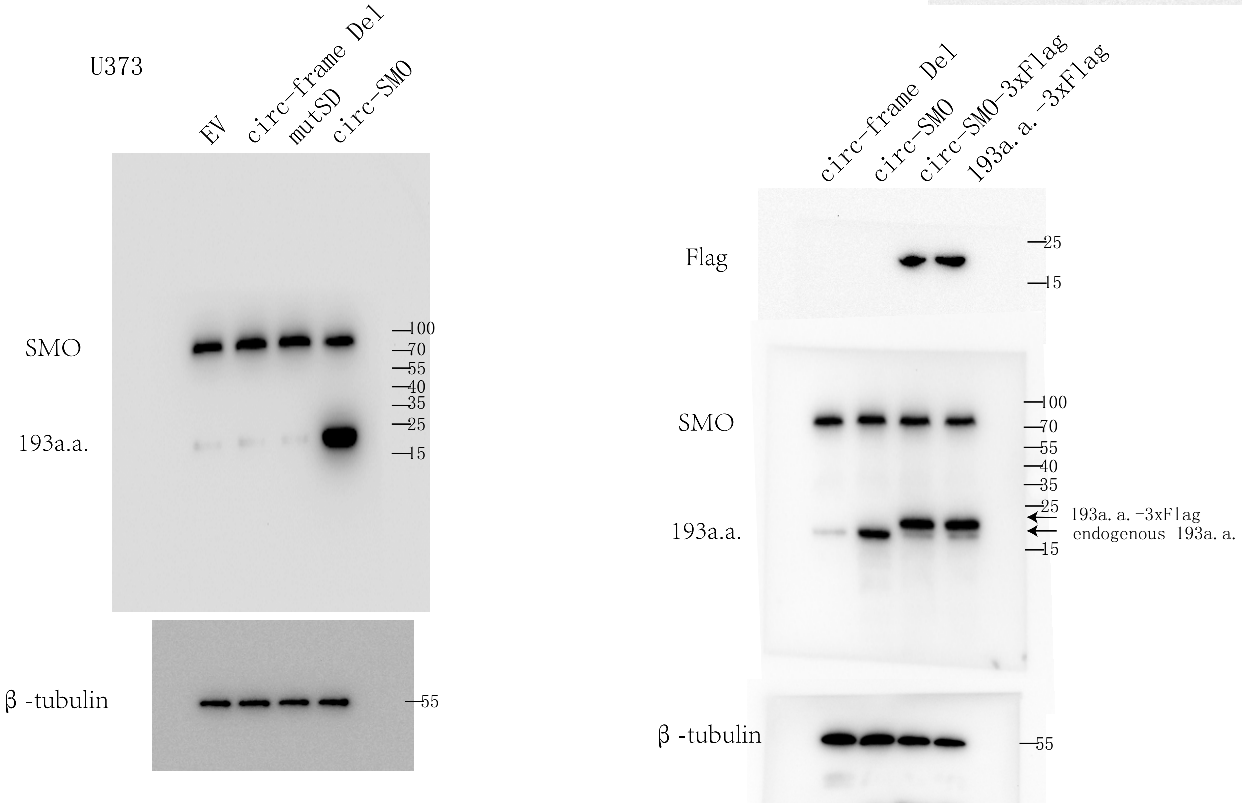

H

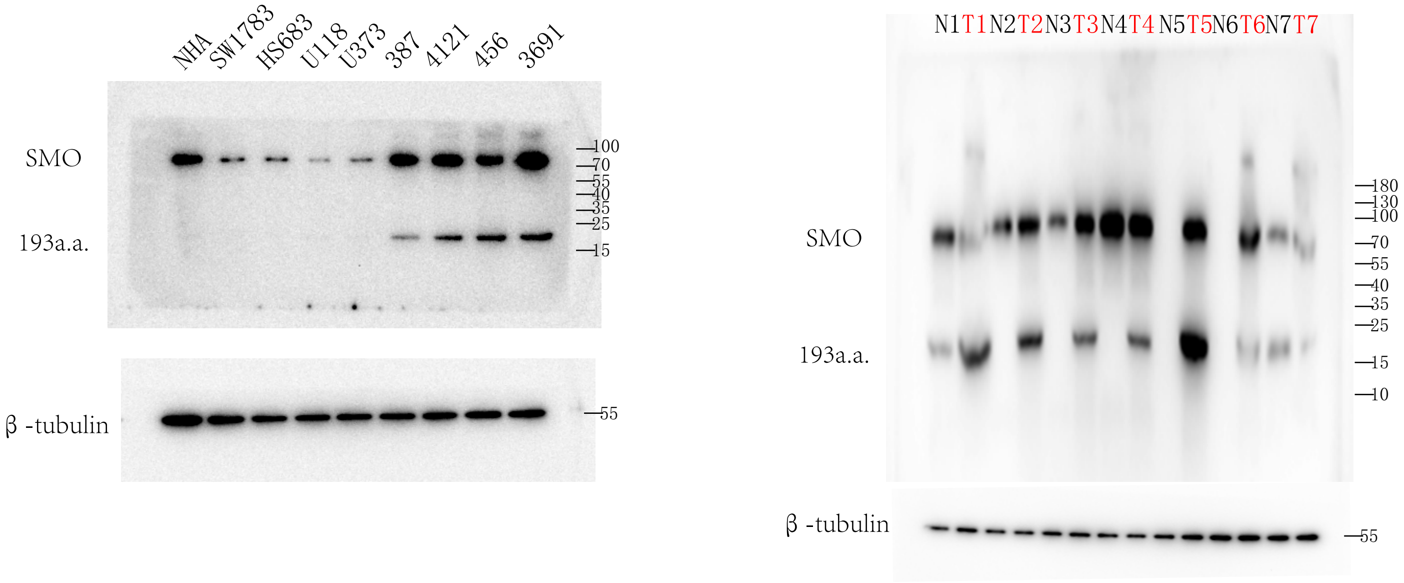

Uncropped Western blots for Figure 3

A

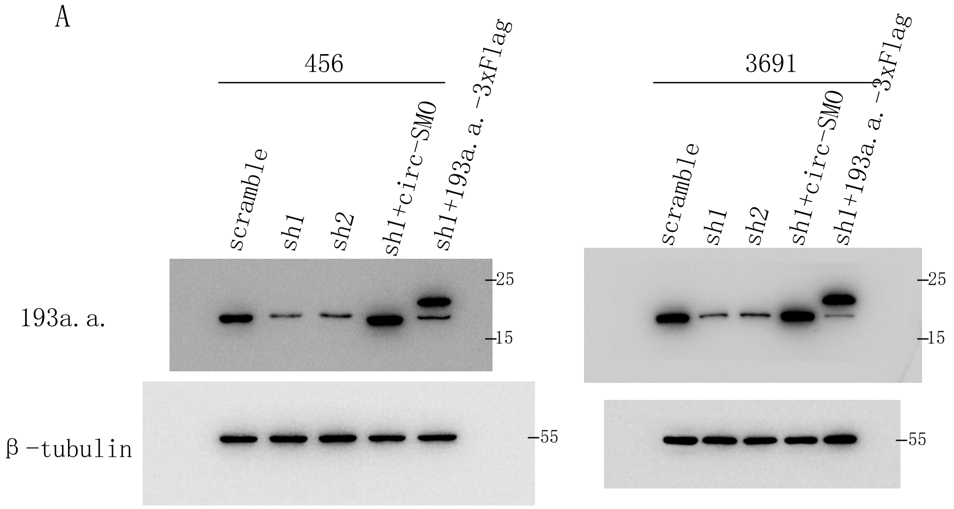

G

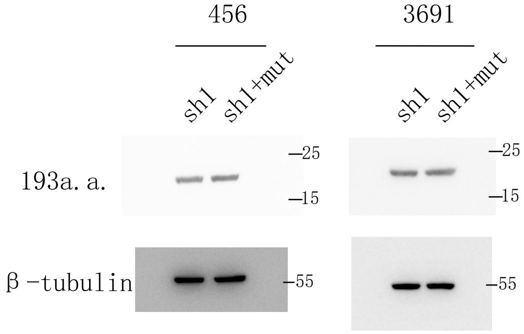

F

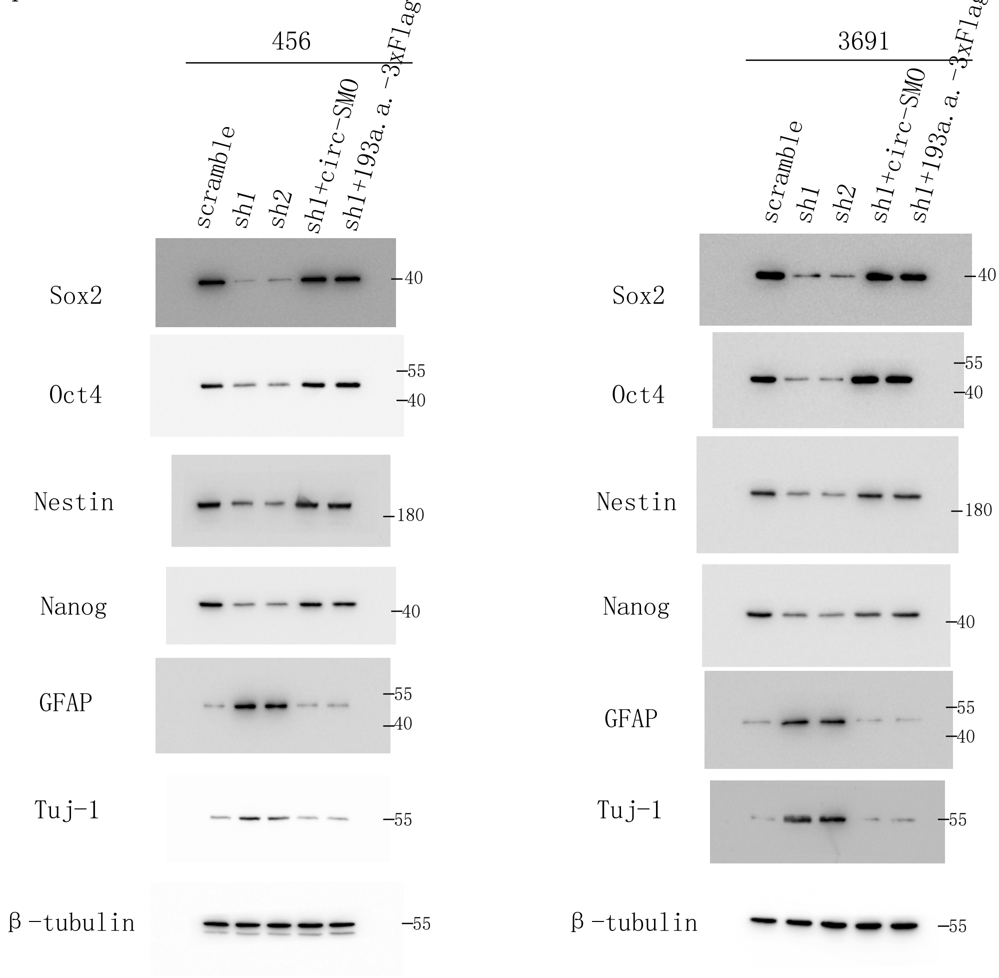

Uncropped Western blots for Figure 4

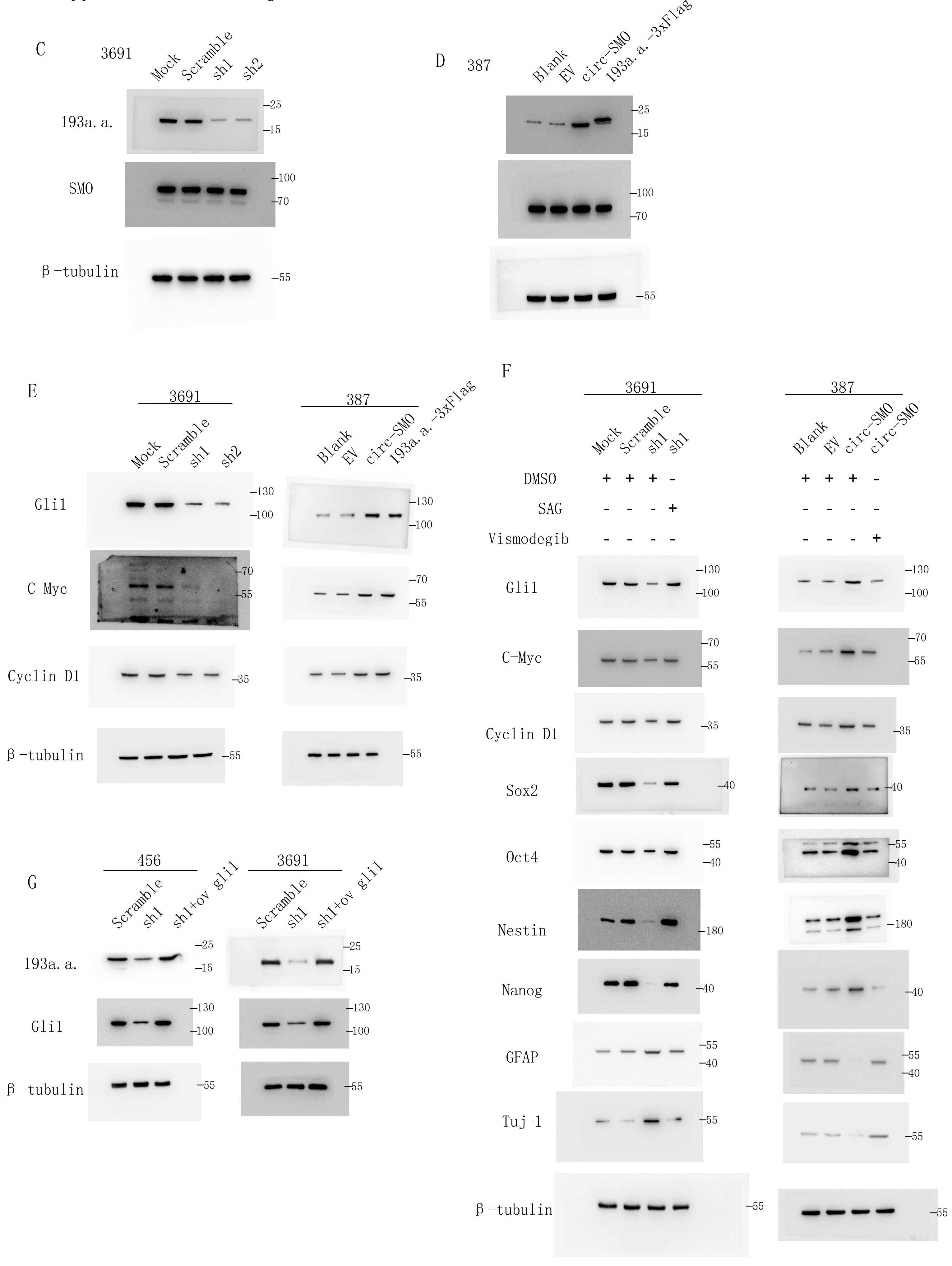

Uncropped Western blots for Figure 5

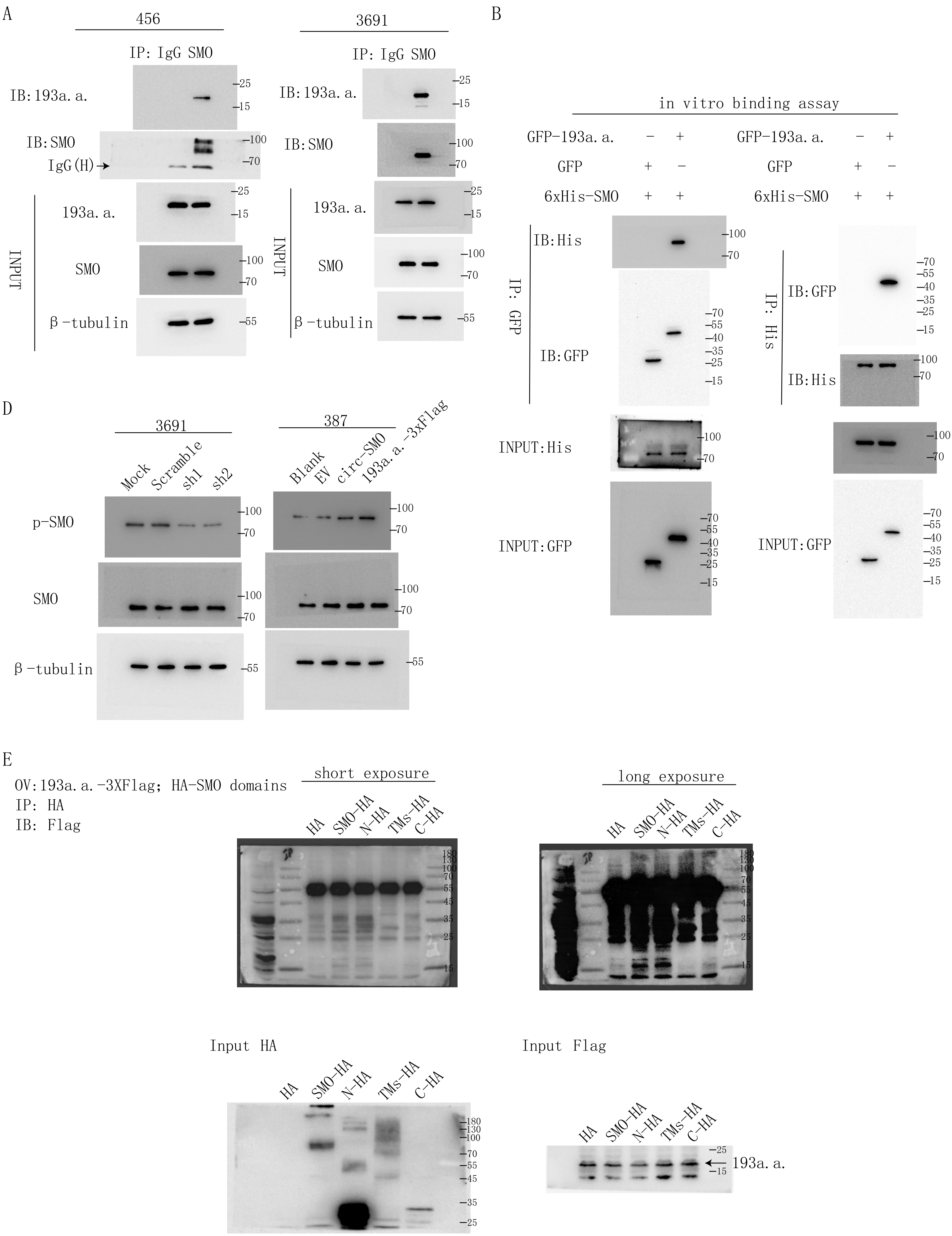

Uncropped Western blots for Figure 5

G

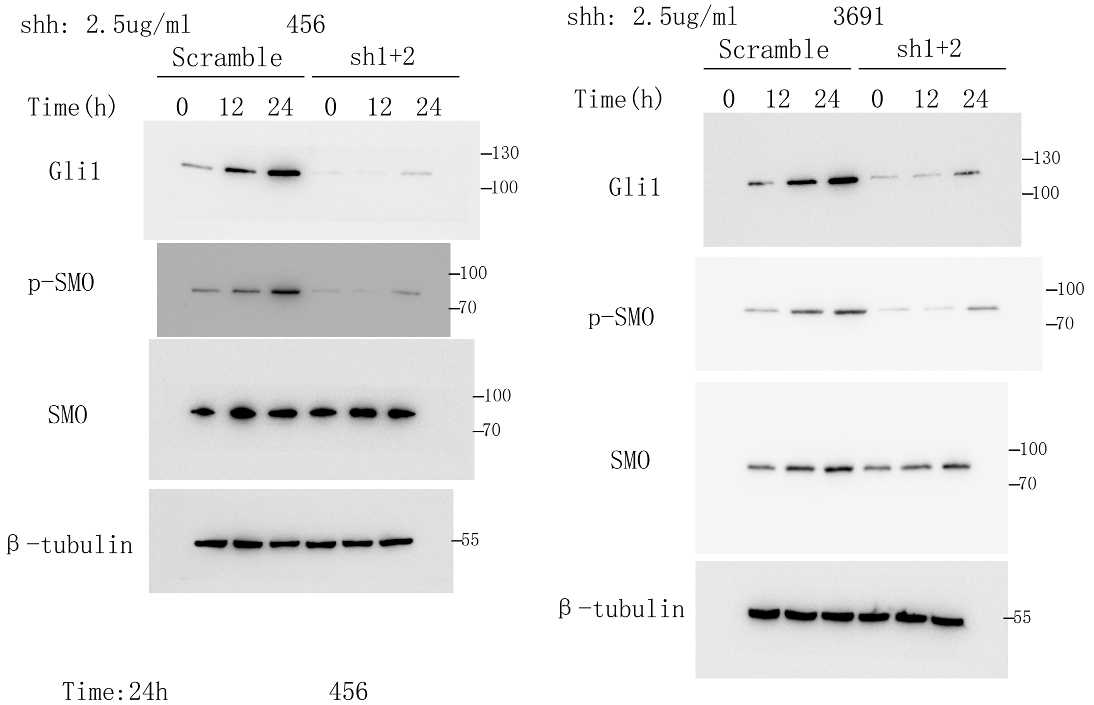

H

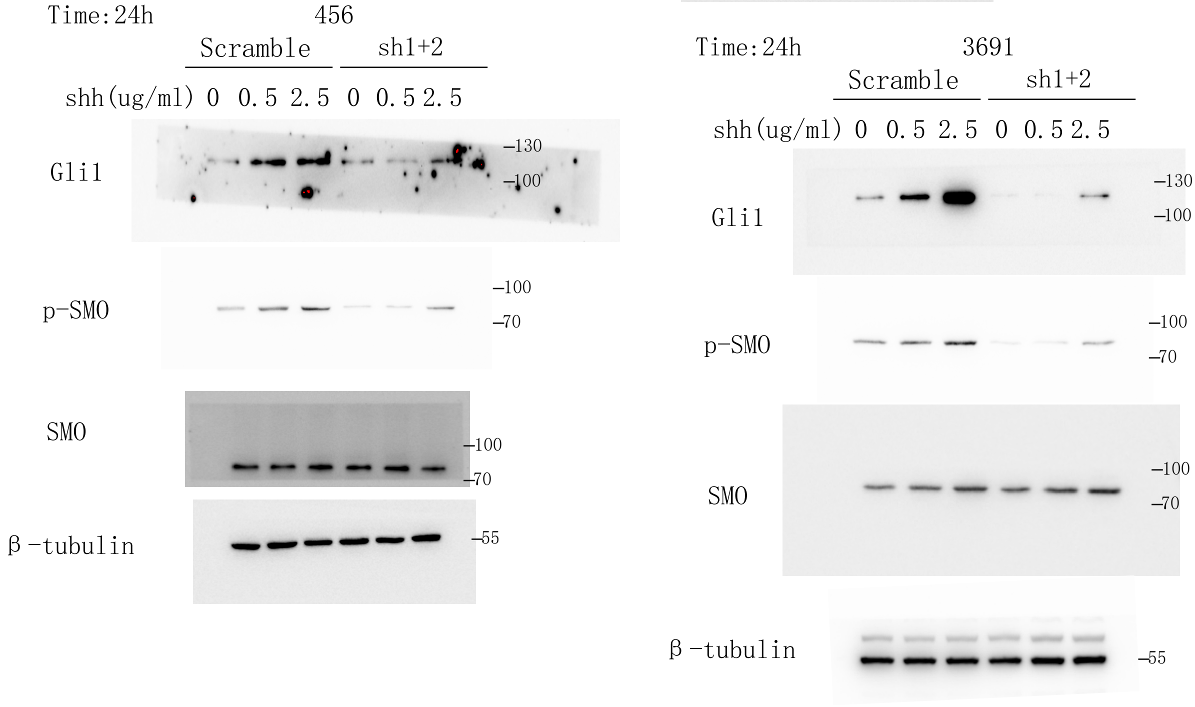

Uncropped Western blots for Figure 6

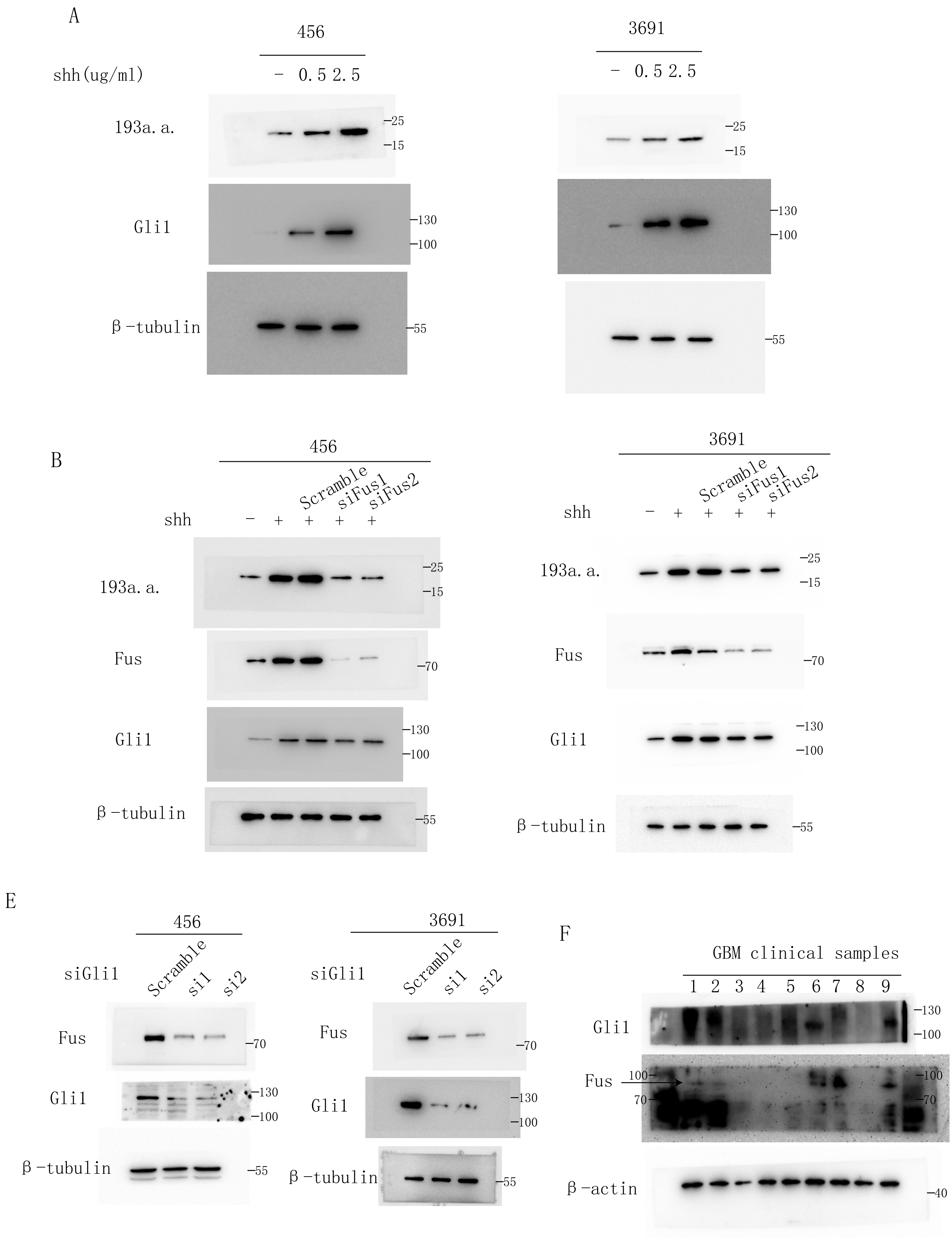

Uncropped Western blots for Fig S2

E

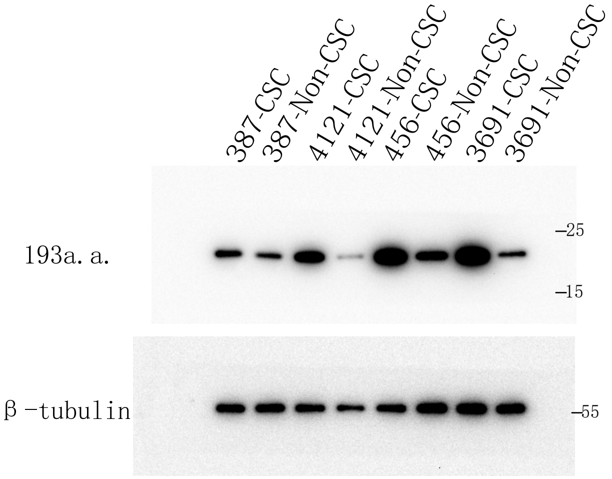

Uncropped Western blots for Fig S3

C

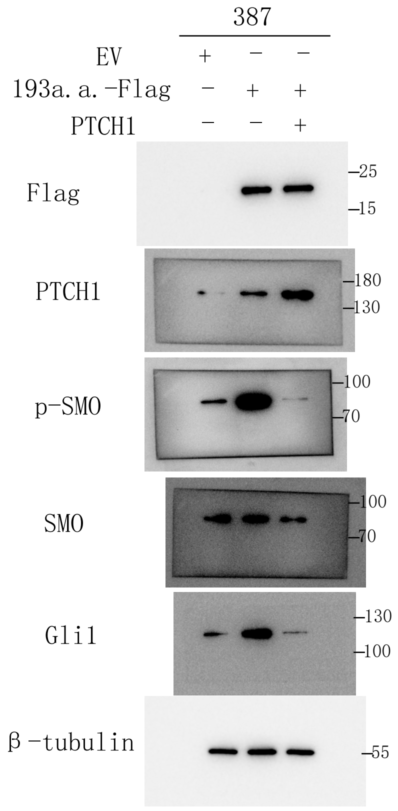

Supplement: Supplementary file 7 — Additional file 7: Uncropped Northern and Western blots. [file 13059_2020_2250_MOESM7_ESM.pdf]
