# Supplementary material for: A novel protein encoded by circular SMO RNA is essential for Hedgehog signaling activation and glioblastoma tumorigenicity
Source: Genome Biol. 2021 Jan 14;22:33. doi: 10.1186/s13059-020-02250-6 (PMC7807754; doi:10.1186/s13059-020-02250-6)
Supplement: Supplementary file 8 — Additional file 8: Review history. [file 13059_2020_2250_MOESM8_ESM.docx]

**Review History**

**First round of review**

**Reviewer 1**

**Are you able to assess all statistics in the manuscript, including the appropriateness of statistical tests used?** No, I do not feel adequately qualified to assess the statistics.

**Comments to author:**

In the manuscript "A Circular SMO RNA Encoded Novel Protein is Essential for Hedgehog Signaling Activation and Glioblastoma Tumorigenicity" by Xujia et al, the authors reported a novel protein (SMO-193a.a.) encoded by circ-SMO, which directly interacts with SMO and promote its activation. Moreover, deprivation of SMO-193a.a. in BTICs suppressed the self-renewal, proliferation in vitro and tumorigenicity in vivo. It is an interesting working, however, there are several issues should be addressed and the paper lacks some technical details. The key problem is that they did not convincingly prove that the short 193aa isoform is indeed produced from circRNA, because the MS data can also come from canonical proteins encoded by linear mRNA, and antibody can also recognize both isoforms. The circRNA-coded SMO-193a.a only differ by a single amino acid from the "host" protein, which makes it hard to separate it from an truncated isoform encode by an alternatively spliced linear SMO RNA. Thus the RNAi experiments will be critical, but the information on the sequence is missing (I assume the shRNA is against the junction, but there is not much room for two shRNAs). The authors should use shRNA against the linear isoform as controls rather than the scramble RNAi, as they need to rule out the possibility that observed effects are due to knockdown of both isoforms.

Specific concerns:

1. Since most of circRNAs are expressed at extremely low levels, RNase R treatment are usually performed to enrich circRNAs. For studies using RiboMinus/RNase R-treated RNA-seq libraries, the variation of enrichment coefficient in the RNase R treatment step may result in biased estimation of circRNA expression levels. In this study (Figure 1B), is the RNA-seq libraries treated with RNase R? The authors described the their circRNA quantification method: "To quantify circRNAs, back-spliced junction reads were scaled to reads per million mapped reads (RPM), and the formula is shown as follows: RPM=10^6C/N. In this formula, C is the number of back-spliced junction reads that uniquely aligned to a circRNA. N is the total number of back-spliced junction reads." For the RNase R treated RNA-seq, this method is not accurate. To reduce false discovery rate, the CIRIquant method will be better (PMID: 31900416).

2. Polysome profiling was used to determine the RNA translation and its efficiency. In Figure 2A, authors analyzed the distribution of circ-SMO in different fractions (Monosome, light polysome and heavy polysome). Most of the wildtype circ-SMO was in the monosome fraction suggests that the translation of circSMO is weak. What is the distribution of wildtype and no-ATG mutated circ-SMO in ribosome free fraction?

3. In figure 2C, authors used circ-luciferase reporter to examine the IRES activity of the circ-SMO. What is the sequence of this IRES? To demonstrate the translation product from circ-SMO (Figure 2E), authors used a circularization frame deletion vector (circ-frame Del) as negative control. However, this vector did not include the flanking introns which may contain unknown IRES to initiate the translation from linear RNA. The back-splicing donor site mutation should be used to eliminate the possibility that SMO-193a.a. was translated from linear RNA.

**Reviewer 2**

**Are you able to assess all statistics in the manuscript, including the appropriateness of statistical tests used?** Yes, and I have assessed the statistics in my report.

**Comments to author:**

In this manuscript (GBIO-D-20-00728), Wu, Xiao, Zhang et al. identify a novel circular RNA of the sonic hedgehog (Shh) family member, smoothened (SMO) of 193 amino acids, that drives self-renewal of glioblastoma (GBM) cancer stem cells (CSCs, although the authors refer to these cells as BTICs). Mechanistically, transcription of this circular RNA is driven by FUS and the circular RNA is present in human GBM tissue and appears to be a stronger biomarker for survival, compared to SMO itself. The studies are generally done in a rigorous manner and make use of elegant mutants and rescue conditions along with a custom-made antibody. Overall, this is an exciting and timely message that is likely to be very well received by the research community. However, there are some narrative/presentation issues and additional studies that would enhance the impact of this manuscript.

1. The claim that Shh is a driver in GBM is not supported by the literature, especially in CSCs as down in one of the first reports (Bar et al., Stem Cells, 2007, PMID 17628016 [NOT CITED]) where Gli activity was only present in a subset of GBM tumors. This should be reflected in the presentation of the manuscript as Shh is likely to be a driver in a subset of GBMs.

2. It is interesting that BTIC models had increase Shh activity, could this be due to culture conditions as the other cell lines are not grown in a similar manner/media conditions. In addition, I would encourage the authors to use the term CSCs, as opposed to BTICs, and think about interrogating SMO-193aa expression between CSCs and non-CSCs (either by enriching for CSCs with enhanced self-renewal capacity or differentiating CSCs to generate non-CSC progeny). This distinction would be useful to make given the functional data provided that links SMO-193aa to CSC maintenance.

3. For the CSC assays, namely in vitro limiting dilution analyses, these should be shown as bar graphs of the inverse of stem cell frequency for easier interpretation (1:2 would be 0.5 and 1:4 would be 0.25) and the stem cell frequencies should be mentioned in the narrative. The current plots could be placed in the supplemental figures.

4. It is unclear by the 373 model was used for Shh signaling assessments (RNA-seq/bioinformatics) when CSC models were used for the functional studies. These studies would be worth repeating with the same models used for in vitro and in vivo functional analyses.

5. For the in vivo studies, it would be useful to assess the tumors themselves. Are there any major changes in proliferation, cell death, CSC markers? Histology could be used here.

6. It would be useful to include a schematic model of the findings to help the reader appreciate the key findings of this manuscript. This could also be mentioned in the 1st paragraph in the discussion.

MINOR

7. The focus of SMO-193aa based on the screening (as it is not the top hit) is a bit odd, given the manuscript 1st focuses on Shh signaling in GBM. This could be better clarified in the narrative to just focus on the top hit in the Shh signaling pathway?

8. In the introduction, it would be worth a brief mention of circular RNAs in GBM and a further expansion/summary in the discussion

9. In Fig. 1I, the number of patients (adjacent versus GBM should be put directly on the figure) and the median survival (KM curve, right) should be places directly on the figure.

10. In Fig. 6F, the correlation is really not that impressive and it almost appears as if there are 2 FUS populations. It may be worth not showing these data as they are confusing. If possible, is survival based on compound FUS/GLI1 more informative?

**Authors Response**

**Point-by-point responses to the reviewers’ comments:**

***Reviewer #1:*** In the manuscript "A Circular SMO RNA Encoded Novel Protein is Essential for Hedgehog Signaling Activation and Glioblastoma Tumorigenicity" by Xujia et al, the authors reported a novel protein (SMO-193a.a.) encoded by circ-SMO, which directly interacts with SMO and promote its activation. Moreover, deprivation of SMO-193a.a. in BTICs suppressed the self-renewal, proliferation in vitro and tumorigenicity in vivo. It is an interesting working, however, there are several issues should be addressed and the paper lacks some technical details.

*The key problem is that they did not convincingly prove that the short 193aa isoform is indeed produced from circRNA, because the MS data can also come from canonical proteins encoded by linear mRNA, and antibody can also recognize both isoforms. The circRNA-coded SMO-193a.a only differ by a single amino acid from the "host" protein, which makes it hard to separate it from an truncated isoform encode by an alternatively spliced linear SMO RNA. Thus the RNAi experiments will be critical, but the information on the sequence is missing (I assume the shRNA is against the junction, but there is not much room for two shRNAs). The authors should use shRNA against the linear isoform as controls rather than the scramble RNAi, as they need to rule out the possibility that observed effects are due to knockdown of both isoforms.*

**Response:**
We thank the reviewer for this excellent question. We provided the shRNA sequences to circ-SMO, as shown in **revised Figure 2E, upper**. shRNAs to linear SMO were also shown. Specific knocking down circ-SMO had no effects on SMO protein level but drastically reduced SMO-193a.a. expression in the same WB (**revised Figure 2E, lower**). Knocking down linear SMO (more than 80%) had a minor effect on SMO-193aa. If the 193a.a. protein was from linear isoforms, there knocking down effects of SMO and circ-SMO should be synchronized. Most likely, this was due to that SMO deprivation reduced the expression of Gli1, which could positively regulate SMO-193a.a. (**Figure 4G**). We admitted that the single amino acid in SMO-193a.a. are hard to detected by Mass Spectra. However, all reported SMO linear isoforms (see below) could not generated SMO-193a.a. protein.

In a recently published paper (*An Insulin-Sensitive Circular RNA that Regulates Lifespan in Drosophila, Mol Cell. 2020 Jul 16;79(2):268-279*), circSfl also encoded a novel protein which had only one different amino acid from the host protein and missed by the Mass Spectra. Besides, our mutated vectors in **revised** **Figure 2F** also excluded the possibility that SMO-193a.a. was from linear isoforms (mutSD and circ-frame Del vectors could not generate SMO-193a.a.). In Northern blot (**Figure 1E**), there were not obviously diminished bands around circ-SMO after RNase R treatment, supported that there were no unknown linear isoforms at this size. With all these evidence, we concluded that SMO-193a.a. was translated from circ-SMO is the most reasonable explanation.

**Specific concerns:**

1. *Since most of circRNAs are expressed at extremely low levels, RNase R treatment are usually performed to enrich circRNAs. For studies using RiboMinus/RNase R-treated RNA-seq libraries, the variation of enrichment coefficient in the RNase R treatment step may result in biased estimation of circRNA expression levels. In this study (Figure 1B), is the RNA-seq libraries treated with RNase R? The authors described the their circRNA quantification method: "To quantify circRNAs, back-spliced junction reads were scaled to reads per million mapped reads (RPM), and the formula is shown as follows: RPM=10^6C/N. In this formula, C is the number of back-spliced junction reads that uniquely aligned to a circRNA. N is the total number of back-spliced junction reads." For the RNase R treated RNA-seq, this method is not accurate. To reduce false discovery rate, the CIRIquant method will be better (PMID: 31900416).*

**Response:**
We thank the reviewer for insightful comments. The RNA-seq libraries were treated with RNase R to enrich circRNAs. We used the CIRIquant method to re-analyzed our data and provided the **revised Figure 1B, revised Figure S1A, B and Table S1**. Related manuscripts were also changed accordingly.

2. *Polysome profiling was used to determine the RNA translation and its efficiency. In Figure 2A, authors analyzed the distribution of circ-SMO in different fractions (Monosome, light polysome and heavy polysome). Most of the wildtype circ-SMO was in the monosome fraction suggests that the translation of circSMO is weak. What is the distribution of wildtype and no-ATG mutated circ-SMO in ribosome free fraction?*

**Response:**
We thank the reviewer for this excellent question. In **revised Figure 2A**, we collected the ribosome free fractions and repeated these experiments. Circ-SMO noATG mutation was enriched in these free fractions. Circ-SMO was enriched in monosome and light polysome fractions. These results were similar with a published article (*Circ-ZNF609 Is a Circular RNA that Can Be Translated and Functions in Myogenesis. Mol Cell. 2017 Apr 6;66(1):22-37*) and consistent with our previous conclusions.

3. *In figure 2C, authors used circ-luciferase reporter to examine the IRES activity of the circ-SMO. What is the sequence of this IRES? To demonstrate the translation product from circ-SMO (Figure 2E), authors used a circularization frame deletion vector (circ-frame Del) as negative control. However, this vector did not include the flanking introns which may contain unknown IRES to initiate the translation from linear RNA. The back-splicing donor site mutation should be used to eliminate the possibility that SMO-193a.a. was translated from linear RNA.*

**Response:**
We thank the reviewer for these excellent questions. The IRES sequences was from position **367-515**(start-end, R score 1.630888, AGGCTTGGGGAGCCCACCTCCAATGAGACTCTGTCCTGCGTCATCATCTTTGTCATCGTGTACTACGCCCTGATGGCTGGTGTGGTTTGGTTTGTGGTCCTCACCTATGCCTGGCACACTTCCTTCAAAGCCCTGGGCACCACCTACCA). The prediction website was http://reprod.njmu.edu.cn/cgi bin/circrnadb/detail_info.php?circ_id=hsa_circ_13808.

The circ-SMO ORF was started at position **151**. Given that started site is before the predicted IRES, translation can only be initiated by the circular reading frame.

To further address this concern, we mutated the back-splicing donor (mutSD) and tested SMO-193a.a. expression. In **revised Figure 2F**, neither mutSD nor circ-frame Del can translate SMO-193a.a.. These results supported that SMO-193a.a. was translated from circ-SMO, instead of linear isoforms.

***Reviewer #2***: In this manuscript (GBIO-D-20-00728), Wu, Xiao, Zhang et al. identify a novel circular RNA of the sonic hedgehog (Shh) family member, smoothened (SMO) of 193 amino acids, that drives self-renewal of glioblastoma (GBM) cancer stem cells (CSCs, although the authors refer to these cells as BTICs). Mechanistically, transcription of this circular RNA is driven by FUS and the circular RNA is present in human GBM tissue and appears to be a stronger biomarker for survival, compared to SMO itself. The studies are generally done in a rigorous manner and make use of elegant mutants and rescue conditions along with a custom-made antibody. Overall, this is an exciting and timely message that is likely to be very well received by the research community. However, there are some narrative/presentation issues and additional studies that would enhance the impact of this manuscript.

**1.** *The claim that Shh is a driver in GBM is not supported by the literature, especially in CSCs as down in one of the first reports (Bar et al., Stem Cells, 2007, PMID 17628016 [NOT CITED]) where Gli activity was only present in a subset of GBM tumors. This should be reflected in the presentation of the manuscript as Shh is likely to be a driver in a subset of GBMs.*

**Response:**
We thank the reviewer for these excellent suggestions. We added these citations in our revised MS with further discussions.

**2.** *It is interesting that BTIC models had increase Shh activity, could this be due to culture conditions as the other cell lines are not grown in a similar manner/media conditions. In addition, I would encourage the authors to use the term CSCs, as opposed to BTICs, and think about interrogating SMO-193aa expression between CSCs and non-CSCs (either by enriching for CSCs with enhanced self-renewal capacity or differentiating CSCs to generate non-CSC progeny). This distinction would be useful to make given the functional data provided that links SMO-193aa to CSC maintenance.*

**Response:**
We thank the reviewer for these insightful comments. We used standard CSC culture conditions (without Shh). BTICs was changed to CSC in the revised MS. To exclude misunderstanding, we unified the cell model to CSC in all functional and animal assays. We also used non-CSCs (CD133 sorting) to compare the SMO-193aa expression. As shown in revised **Figure S2E**, CD133 negative non-CSCs had lower level of SMO-193a.a. than CD133 positive CSCs.

**3.** *For the CSC assays, namely in vitro limiting dilution analyses, these should be shown as bar graphs of the inverse of stem cell frequency for easier interpretation (1:2 would be 0.5 and 1:4 would be 0.25) and the stem cell frequencies should be mentioned in the narrative. The current plots could be placed in the supplemental figures.*

**Response:**
We thank the reviewer for these excellent suggestions. We had revised these results in **Figure 3, 4**.

**4.** *It is unclear by the 373 model was used for Shh signaling assessments (RNA-seq/bioinformatics) when CSC models were used for the functional studies. These studies would be worth repeating with the same models used for in vitro and in vivo functional analyses.*

**Response:**
We thank the reviewer for these excellent suggestions. We used 373 model due to its lower SMO-193a.a. level. In the **revised Figure 4A, Figure S3A and Table S2-5**, CSCs with shRNAs were used for RNA-seq and bioinformatics. The new data was consistent with previous conclusions.

**5.** *For the in vivo studies, it would be useful to assess the tumors themselves. Are there any major changes in proliferation, cell death, CSC markers? Histology could be used here.*

**Response:**
We thank the reviewer for these excellent suggestions. We performed IHC in **revised Figure S4C** and assessed several markers as suggested by the reviewer.

**6.** *It would be useful to include a schematic model of the findings to help the reader appreciate the key findings of this manuscript. This could also be mentioned in the 1st paragraph in the discussion.*

**Response:**
We thank the reviewer for this excellent suggestion. A graphic abstract was added to **Figure 6J** and descried in the discussion.

**MINOR**

**7.** *The focus of SMO-193aa based on the screening (as it is not the top hit) is a bit odd, given the manuscript 1st focuses on Shh signaling in GBM. This could be better clarified in the narrative to just focus on the top hit in the Shh signaling pathway?*

**Response:**
We thank the reviewer for this excellent suggestion. We had revised our MS accordingly.

**8.** *In the introduction, it would be worth a brief mention of circular RNAs in GBM and a further expansion/summary in the discussion*

**Response:**
We thank the reviewer for this excellent suggestion. We had revised our MS accordingly.

**9.** *In Fig. 1I, the number of patients (adjacent versus GBM should be put directly on the figure) and the median survival (KM curve, right) should be places directly on the figure.*

**Response:**
We thank the reviewer for this excellent suggestion. We had revised **Figure 1I** accordingly.

**10.** *In Fig. 6F, the correlation is really not that impressive and it almost appears as if there are 2 FUS populations. It may be worth not showing these data as they are confusing. If possible, is survival based on compound FUS/GLI1 more informative?*

**Response:**
We thank the reviewer for this excellent suggestion. We had **revised** **Figure 6F** accordingly. The confusing parts were removed. Compound FUS/Gli1 analysis was more informative in in LGG but not GBM. We did not include this result in the revised manuscript.

**Second round of review**

**Reviewer 1**

In their revised manuscript, the authors have addressed most of my concerns with additional experiments, but there remain some doubts on the translation of circSMO.

First of all, Fig 1E has many extra bands, even after the treatment of Rnase R, casting a lots of doubt on the identity of each band.

Also in Fig.1, the authors showed that circ-SMO expression level was similar or even lower than SMO mRNA, however the protein level of circ-SMO was slightly lower than SMO (Fig 2D and E). Why such low translation efficiency of circ-SMO can produce large amount of circ-SMO proteins?

In addition, the authors repeated the polysome profiling experiments and confirmed that circ-SMO was enriched in monosome and light polysome fractions, while SMO mRNA was enriched in heavy polysome fractions (Fig 2A), suggesting that the translation of circ-SMO was much lower than SMO mRNA. Therefore it seems contradictory that the circ-SMOs with low translation efficiency (Fig 2A) can generate so many translation products that are comparable to the linear forms (Fig 2D). This is against the current knowledge of on cap-independent translation.

**Reviewer 2**

The authors have done a great job at addressing all previously raised issues. There are a series of minor issues that should be addressed prior to publication.

1. S. Fig. 2B, the sphere formation graphs should be presented as bar graphs (was revised for Figs. 3, 4).

2. S. Fig. 2E, "CSC", "non'CSCs" should be clearly indicated on the blots, not symbols as this in confusing.

4. S. Fig. 4, the staining provided should be quantified (and accompanied by bar graphs).

**Authors Response**

**Point-by-point responses to the reviewers’ comments:**

*Reviewer #1: In their revised manuscript, the authors have addressed most of my concerns with additional experiments, but there remain some doubts on the translation of circSMO. First of all, Fig 1E has many extra bands, even after the treatment of Rnase R, casting a lots of doubt on the identity of each band.*

Response

We thank the reviewer for this insightful comment. To lower the background of Northern Blot, we reduced the loading amount of total RNA in revised Figure 1E. To further indicate circ-SMO, we also used junction specifical shRNAs to circ-SMO. The same size of diminished or overexpressed circ-SMO also supported the specificity of recognizing.

*Also in Fig.1, the authors showed that circ-SMO expression level was similar or even lower than SMO mRNA, however the protein level of circ-SMO was slightly lower than SMO (Fig 2D and E). Why such low translation efficiency of circ-SMO can produce large amount of circ-SMO proteins? In addition, the authors repeated the polysome profiling experiments and confirmed that circ-SMO was enriched in monosome and light polysome fractions, while SMO mRNA was enriched in heavy polysome fractions (Fig 2A), suggesting that the translation of circ-SMO was much lower than SMO*

*mRNA. Therefore it seems contradictory that the circ-SMOs with low translation efficiency (Fig 2A) can generate so many translation products that are comparable to the linear forms (Fig 2D). This is against the current knowledge of on cap-independent translation.*

Response

We thank the reviewer for this insightful comment. We have several lines of evidence

to explain our results.

1.In Fig.2D, 373 cells had very low endogenous SMO-193a.a..The right lane was overexpression with circ-SMO. 3691 cells had comparable level of SMO mRNA and circ-SMO (see northern in Fig. 1E), but endogenous circ-SMO was also lower expressed than SMO.

2.A previous report indicated that circRNA could be efficiently translated as a comparable level of mRNA(1). As shown in below (Fig. 6 of that paper), circRNA could stably generate proteins in 6 days. Although the circRNA was not an endogenous one, the longer translation time of circRNA was highly implied.

3.Besides, Biever et al. reported that monosome-preferring transcripts encoded proteins that span a broad range of abundances in the neuropil(2). Their data indicated that the ribosome footprint density throughout the transcript ORF in the neuropil monosomes (Fig. 2B of that paper, see below).

Above information all suggested that circRNA in neuro-system could efficiently translate proteins.

To further validate our results, we first detected SMO-193a.a. and SMO half-life in 3691 BTICs. Given the comparable level of circ-SMO/SMO mRNA and their protein products, endogenous half-life of SMO-193a.a. and SMO was determined by adding CHX. SMO-193a.a. and SMO had no significant different half-life (see below).

Next, we transfect circ-SMO-Flag and SMO-Flag plasmid into 373 cells and detected the Flag-tag protein level at different time point. Compare to SMO-Flag, translation of circ-SMO-Flag was more durable (see below). This may be due to that circ-SMO had a higher stability than SMO mRNA (our manuscript, Figure 1D).

Further evidence including that m6A could promote translation of circRNA(3). CircSMO distribution in ribosome fragments was in accordance with those m6A driven coding-circRNAs (Figures of that paper).

We also found three high confidence m6A sites based on circ-SMO sequence searching (see below). The m6A driven translation also could be the reason of high SMO-193a.a. level in 3691 BTICs.

Based on above all evidence, we concluded that 1. Circ-SMO was high in BTICs; 2.

Although translation efficiency of circ-SMO maybe lower than SMO mRNA, the higher stability of circ-SMO and higher monosome elongation efficiency in neuro system supported SMO-193a.a. expression; 3. Circ-SMO could also be driven by m6A to translate protein.

We think our main conclusion was fully supported by current evidence and we hope our revised manuscript could meet the approved by the reviewer. You kindly input on our manuscript will be very much appreciated.

Reference

1.Wesselhoeft RA, Kowalski PS, Anderson DG. Engineering circular RNA for potent

and stable translation in eukaryotic cells. Nat Commun. 2018;9(1):2629.

2.Biever A, Glock C, Tushev G, Ciirdaeva E, Dalmay T, Langer JD, et al. Monosomes

actively translate synaptic mRNAs in neuronal processes. Science. 2020;367(6477).

3.Yang Y, Fan X, Mao M, Song X, Wu P, Zhang Y, et al. Extensive translation of

circular RNAs driven by N(6)-methyladenosine. Cell Res. 2017;27(5):626-41.

**Third round of review**

**Reviewer 1**

The authors have made a lot of effort to address my comments, which is much appreciated. Although I still think they did not have a very strong evidence to support that the small isoform is indeed translated from circRNAs, many of their data support this conclusion indirectly. I am OK to give the authors a benefit of doubt and let the paper pass.

However, I think the author should use their original picture in fig. 1E, because reducing the loading amount may help "hiding" some meaningful data despite it makes the picture looks better (more clean).

In addition, if they think m6A play a role in driving circSMO translation, they should discuss the data in the main text, and probably should test it with over-expression of m6a eraser.

**Authors Response**

**Point-by-point responses to the reviewers’ comments:**

**Reviewer #1**:

*However, I think the author should use their original picture in fig. 1E, because reducing the loading amount may help "hiding" some meaningful data despite it makes the picture looks better (more clean).*

**Response:**

We thank the reviewer for this insightful comment. We used the original Northern blots in the **Revised Fig 1E**.

*In addition, if they think m6A play a role in driving circSMO translation, they should discuss the data in the main text, and probably should test it with over-expression of m6a eraser.*

**Response:**

We thank the reviewer for this insightful comment. We have discussed the data related to the role of m6A in driving circ-SMO translation in the revised manuscript and **Revised Fig. S2B, C**.
